# Global colistin use: a review of the emergence of resistant *Enterobacterales* and the impact on their genetic basis

**DOI:** 10.1093/femsre/fuab049

**Published:** 2021-10-06

**Authors:** Ulrike Binsker, Annemarie Käsbohrer, Jens A Hammerl

**Affiliations:** Unit Epidemiology, Zoonoses and Antimicrobial Resistance, Department Biological Safety, German Federal Institute for Risk Assessment, Diedersdorfer Weg 1, 12277 Berlin, Germany; Unit Epidemiology, Zoonoses and Antimicrobial Resistance, Department Biological Safety, German Federal Institute for Risk Assessment, Diedersdorfer Weg 1, 12277 Berlin, Germany; Department for Farm Animals and Veterinary Public Health, Institute of Veterinary Public Health, University of Veterinary Medicine Vienna, Veterinärplatz 1, 1210 Vienna, Austria; Unit Epidemiology, Zoonoses and Antimicrobial Resistance, Department Biological Safety, German Federal Institute for Risk Assessment, Diedersdorfer Weg 1, 12277 Berlin, Germany

**Keywords:** polymyxin, antimicrobial use, chromosome, One Health, *mcr*, lipid A

## Abstract

The dramatic global rise of MDR and XDR *Enterobacterales* in human medicine forced clinicians to the reintroduction of colistin as last-resort drug. Meanwhile, colistin is used in the veterinary medicine since its discovery, leading to a steadily increasing prevalence of resistant isolates in the livestock and meat-based food sector. Consequently, transmission of resistant isolates from animals to humans, acquisition via food and exposure to colistin in the clinic are reasons for the increased prevalence of colistin-resistant *Enterobacterales* in humans in the last decades. Initially, resistance mechanisms were caused by mutations in chromosomal genes. However, since the discovery in 2015, the focus has shifted exclusively to mobile colistin resistances (*mcr*). This review will advance the understanding of chromosomal-mediated resistance mechanisms in *Enterobacterales*. We provide an overview about genes involved in colistin resistance and the current global situation of colistin-resistant *Enterobacterales*. A comparison of the global colistin use in veterinary and human medicine highlights the effort to reduce colistin sales in veterinary medicine under the One Health approach. In contrast, it uncovers the alarming rise in colistin consumption in human medicine due to the emergence of MDR *Enterobacterales*, which might be an important driver for the increasing emergence of chromosome-mediated colistin resistance.

## Teaser

Colistin resistance in *Enterobacterales* is highly complex and mutations in chromosomally encoded genes are of great concern due to the use of colistin as last-line drug.

## INTRODUCTION

Antimicrobial resistance (AMR) is recognized as one of the greatest challenges for human health worldwide. Over-prescribing and over-using of antibiotics in human and veterinary medicine has led to the development of multidrug-resistant (MDR; at least one antimicrobial agent in three or more antibiotic classes), extensively drug-resistant (XDR; species are only susceptible to two antimicrobial drug classes) and pandrug-resistant (PDR; resistant to almost all commercially available antimicrobials) Gram-negative bacteria (Magiorakos *et al*. 2012). Rising AMR causes difficult-to-treat infections, therapeutic complications, longer hospital stays and increased mortality. Especially, extended-spectrum beta-lactamase (ESBL)-producing *Enterobacterales*, such as *Escherichia coli* (*E. coli*) and *Enterobacter* spp., as well as carbapenem-resistant *Enterobacterales* (CRE), particularly *Klebsiella* spp., have been increasingly associated with high morbidity rates due to limited treatment options. According to the European Centre for Disease Prevention and Control, more than 670 000 bacterial infections can be attributed to MDR bacteria, which causes 33 000 death annually in Europe (European Centre for Disease Prevention and Control 2019). As a result, it is estimated that MDR infections and complications cost the healthcare system 1.1 billion annually in Europe. The enormous lack of novel antimicrobials active against these MDR Gram-negative bacteria, particularly those producing carbapenemases, requires the growing use of last-resort antibiotics, such as colistin (Falagas and Kasiakou, 2005; Grundmann *et al*. 2017). In contrast, colistin has been continuously used in the global livestock production for prophylactic, therapeutic and even for growth promotion purposes, which has already been banned in Europe since 2006 (European Commission 2005). The frequent application of antibiotics in food-producing animals is associated with selection of resistant zoonotic strains that can be transmitted directly from animal to human or indirectly via the food chain and eventually causing difficult-to-treat diseases in humans (Marshall and Levy, 2011). Therefore, concepts have been developed and implemented to reduce and limit the use of antibiotics in animals and humans. As such, the globally active One Health Commission focuses on the protection of human health by protecting animal and environmental health, biodiversity and food safety. The One Health approach emerged internationally to primarily address emerging and re-emerging zoonotic diseases (Min, Allen-Scott and Buntain 2013). However, when the concept grew, further interdependent areas were included. Nowadays, One Health also aims to address the urgent problem of AMR by reducing the use of antimicrobials in food-producing animals, which means human health and animal health are interconnected. Thus, controlling animal and zoonotic diseases is effective in controlling human diseases.

The present review outlines the current global colistin resistance situation in *Enterobacterales a*nd summarizes the consumption of colistin in the veterinary sector and human medicine in different countries worldwide. The focus of the review was placed on the so far identified bacterial mechanisms mediating colistin resistance highlighting naturally-occurring mutations in chromosomally encoded genes. Since the discovery in 2015, the scientific interest has shifted almost exclusively to plasmid-mediated and transmissible colistin resistance (*mcr*). In contrast to chromosomal-mediated resistance, plasmid-mediated resistance can easily disseminate among different enterobacterial genera, which occurs predominantly in the livestock sector. It can be assumed that with the continuous effort to reduce colistin consumption in veterinary medicine under the One Health approach, the prevalence of *mcr*-bearing plasmids will decrease to a low but stable level in the future. In absence of colistin as selective pressure, bacteria will remove their redundant *mcr*-plasmids due to the energy-consuming replication mechanism of the plasmids. On the other hand, chromosomally mediated colistin resistance is predominantly described in human clinical *Enterobacterale*s isolates and their prevalence will increase especially in human medicine, where colistin is increasingly used as a last resort treatment for carbapenemase-producing pathogens. Chromosomal alterations in the core genome are characterized by a high stability and irreversibility. This may become a significant health problem when chromosomal mutations accumulate in key human pathogenic lineages. Furthermore, this review stresses the urgent need in the routine monitoring of colistin resistance in *Enterobacterales* isolated from human and veterinary niches, the food chain and the environment.

### *Enterobacterales* – a major host for colistin resistance

The family of *Enterobacterales* is a large group of Gram-negative bacteria and members, such as *Escherichia*, *Klebsiella*, *Salmonella*, *Enterobacter*, *Serratia*, *Citrobacter* and *Proteus*, are common inhabitants of the gastrointestinal tract of humans and animals (Guentzel, 1996; Marshall and Levy, 2011). However, due to the ability to acquire and disseminate a wide variety of antibiotic resistances, some members of the *Enterobacterales* family are among the most significant public health problems worldwide (Smet *et al*. 2010; Navon-Venezia, Kondratyeva and Carattoli 2017). In early 2017, the World Health Organization (WHO) published a pathogen priority list, which included CRE as “critical” antibiotic-resistant bacteria that represent an enormous threat to public health (World Health Organization 2017). In addition, in humans, they account for about 80% of Gram-negative isolates with a variety of diseases including urinary tract infections, pneumonia, diarrhea, meningitis, sepsis, endotoxic shock and others (Oliveira and Reygaert, 2021). Comparable to humans, certain pathogenic lineages of, for example *E. coli* and *Salmonella*, can cause infections in animals such as diarrhea and colibacillosis (Catry *et al*. 2015). Colistin resistances have been increasingly described in *Enterobacterales* of human and animal origin (Monaco *et al*. 2014; European Centre for Disease Prevention and Control 2020; Homeier-Bachmann *et al*. 2021). Those resistances were initially thought to be located on the chromosome, until 2015, when the first mobile colistin resistance (*mcr*) gene carried by a plasmid has been found (Liu *et al*. 2016). The occurrence of *mcr*-genes in *Enterobacterales* has been described in detail elsewhere and is outside of the scope of the present review. A short overview is given in the supplementary material and Table S1(Supporting Information).

### Antibacterial effect of colistin

The polymyxins have bactericidal activity against most members of the *Enterobacterales* family including *E. coli*, *Klebsiella*, *Salmonella*, *Shigella* and *Enterobacter*, as well as other clinically relevant Gram-negative pathogens such as *Acinetobacter baumannii* and *Pseudomonas aeruginosa*. On the other hand, the polymyxins demonstrated no activity towards Gram-negative and Gram-positive cocci and Gram-positive bacilli. In addition, polymyxins lack activity against intrinsically resistant species, including *Neisseria*, *Serratia*, *Stenotrophomonas*, *Providencia* and *Proteus* spp., *Burkholderia pseudomallei*, *Morganella morganii* and *Edwardsiella tarda* as well as anaerobic bacteria (Muyembe, Vandepitte and Desmyter 1973; Shimizu, Iyobe and Mitsuhashi 1977; Storm, Rosenthal and Swanson 1977; Pogue *et al*. 2011).

The polyanionic lipopolysaccharide (LPS) structure of Gram-negative bacteria, consisting of a lipid A moiety, a conserved oligosaccharide core (2-keto-3-deoxyoctonoic acid, Kdo) and an O-antigen group, is the main target of colistin. The bactericidal effect of colistin is based on its amphipathicity and a multi-step mechanism. Lipid A produced by most species carries a negative charge due to the presence of free phosphate groups. Divalent cations such as calcium (Ca^2+^) and magnesium (Mg^2+^) stabilize LPS by binding to the phosphate groups (Schnaitman, 1971). Initially, colistin establishes an electrostatic interaction with lipid A. The positively-charged diaminobutyric acid (Dab) residues of colistin bind the negatively charged phosphate groups of lipid A and replace Ca^2+^ and Mg^2+^ ions in a competitive manner thereby destabilizing the LPS and reducing the outer membrane integrity (Velkov *et al*. 2010). Thereafter, the N-terminal fatty acid side chain and the hydrophobic domain of colistin (Leu^6^-L-Leu^7^) insert into the outer membrane, leading to expansion of the lipid monolayer. The increased membrane permeability causes release of periplasmic substances, uptake of colistin into the periplasm, probable integration of the molecule into the inner membrane and eventually cell death (Dixon and Chopra, 1986). Although colistin initially interacts with lipid A, the detailed mechanism of its activity has not yet been fully deciphered and is subject to current investigations (Moffatt, Harper and Boyce 2019).

## RESISTANCE MECHANISMS

### LPS modifying enzymes and their regulators

The composition of LPS is explained very comprehensively by Raetz and colleagues, which is highly heterogeneous and is adapted to environmental stimuli, e.g. pH changes or the presence of cationic antimicrobial peptides. LPS often carries nonstoichiometric substitutions in lipid A and in the inner core. Its heterogeneity is achieved at different position of the LPS structure by (i) modification of lipid A, (ii) changes in the inner core as well as (iii) truncation of the outer core. LPS changes underlie a tight regulation and mediate resistance to cationic antimicrobial peptides, such as colistin (Raetz *et al*. 2007). The interaction of positively charged Dab residues of colistin with the negatively charged phosphoryl-groups of lipid A is pivotal for its bactericidal activity. Consequently, polymyxin resistance is achieved by remodeling of LPS by incorporation of pEtN and L-Ara4N leading to a reduction of the LPS net negative charge by shielding phosphate and carboxyl groups, which in turn impedes binding of polymyxins.

The biosynthesis of L-Ara4N and addition to LPS requires the enzymes Ugd, ArnB, ArnC, ArnA, ArnD, ArnT, ArnE and ArnF. In *E. coli* and *Salmonella* spp., L-Ara4N is preferentially linked to the 4′phosphate group of lipid A by ArnT, but it can also be found at the 1′position (Raetz *et al*. 2007). Opposite to L-Ara4N, pEtN is primarily added to the 1′phosphate group, however, can also be linked to the 4′position, when L-Ara4N is absent (Raetz *et al*. 2007).

The proteins responsible for the regulation, biosynthesis and addition of L-Ara4N and pEtN are chromosomally encoded and respond to the presence of environmental signals or mutational changes. In the following, only those proteins are mentioned for which a genetic alteration could be detected in naturally occurring colistin-resistant animal or human isolates.

### *pmrCAB* operon

The operon of *Enterobacterales* encodes the two-component signal transduction system (TCS) PmrAB (BasRS in *E. coli*) including the phosphoethanolamine transferase PmrC (EptA in *E. coli*), which modifies the 1′-phosphate group of lipid A. Interruption of the regulatory mechanisms due to critical genetic changes provokes constitutive activation of PmrA or PmrB, subsequent overexpression of LPS modifying enzymes and thus colistin resistance.

In total, two hotspots for missense mutations in PmrA (BasR) affecting the amino acids (aa) G53 and R81 within the phosphate receiver (REC) domain have been identified in *Enterobacterales*, of which mutations at position G53 have been experimentally confirmed to mediate colistin resistance (Table 1; Sun *et al*. 2009; Diene *et al*. 2013; Olaitan *et al*. 2014; Quesada *et al*. 2015; Nordmann, Jayol and Poirel 2016; Bourrel *et al*. 2019; Janssen *et al*. 2020). Among different species, the histidine kinase gene *pmrB* seems to be the more common site for gain-of-function mutations compared to the response regulator gene *pmrA*. Hot spots for mutations were located in L14, P94, E121, T156, V161 and G206 affecting the N-terminus, the HAMP and the HisKA domain of the protein (Table 2; Sun *et al*. 2009; Quesada *et al*. 2015; Delannoy *et al*. 2017; Sato *et al*. 2018; Bourrel *et al*. 2019; Kathayat *et al*. 2020). However, only the missense mutation at the position G206 was experimentally confirmed (Sato *et al*. 2018). Notably, mutational studies of EnvZ in *E. coli*, a homolog of PmrB, showed that mutations in the TM1 domain, the HAMP domain and the HisKA domain increase the ratio of kinase activity to phosphatase activity (Park and Inouye, 1997; Hsing *et al*. 1998; Zhu and Inouye, 2002). Thus, it is possible that mutations in the same domains of PmrB would lead to an increased kinase/phosphatase activity and consequently an increased transfer of the phosphate to PmrA.

**Table 1.** Mutations in the response regulator PmrA of *Enterobacterales*.

Bacterial genera	ST	PmrA protein, length [aa]	Protein domain (residues)	Amino acid change	Resulting colistin MICs mg/L	Experimentally confirmed mutation	Bacterial source and comments	Reference
*E. coli*	ns	222 (BasR)	REC (1–112)	G15R	ns	Not confirmed	Human clinical specimen	Bourrel *et al*. (2019)
	ns	222 (BasR)	REC (1–112)	S29G	ns	Not confirmed	Chicken feces	Vounba *et al*. 2019
	ns	222 (BasR)	REC (1–112)	S39I	4	Not confirmed	Swine feces, also mutation in PmrA R81S	Quesada *et al*. (2015)
	131	222 (BasR)	REC (1–112)	G53A	8	Confirmed	Human clinical blood isolate	Janssen *et al*. (2020)
	ns	222 (BasR)	REC (1–112)	G53A or G53C or G53E or G53R or G53S or G53V or G53W	ns	Not confirmed	Human clinical specimen	Bourrel *et al*. (2019)
	ns	222 (BasR)	REC (1–112)	A80V	ns	Not confirmed	Avian pathogenic *E. coli*	Kathayat *et al*. (2020)
	ns	222 (BasR)	REC (1–112)	R81L or R81S	ns	Not confirmed	Human clinical specimen	Bourrel *et al*. (2019)
	131	222 (BasR)	REC (1–112)	L105P	16	Confirmed	Human clinical specimen	Sato *et al*. (2018)
	ns	222 (BasR)	–	G144S	ns	Not confirmed	Diseased pig (also observed in sensitive strains)	Delannoy *et al*. (2017)
*Klebsiella* spp.	ns	223	REC (1–112)	S42N	ns	Not confirmed	Human feces from healthy individuals	Olaitan *et al*. (2014)
	ns	223	REC (1–112)	G53C or G53S	32 / 128	Not confirmed	Human clinical specimen	Nordmann *et al*. (2016)
	ns	223	REC (1–112)	G53C or G53S	ns	Not confirmed	Human feces from healthy individuals	Olaitan *et al*. (2014)
	ns	223	REC (1–112)	E57G	ns	Not confirmed	Human clinical isolate, also mutation in PmrB T246A	Samuelsen *et al*. (2017)
	ns	223	REC (1–112)	D86E	>8	Not confirmed	Human clinical isolate, also mutation in PmrA G35C	Samuelsen *et al*. (2017)
*Enterobacter* spp.	ns	222	REC (1–112)	G53S	> 16	Confirmed	Human clinical specimen	Diene *et al*. (2013)
	ns	222	REC (1–112)	S64C	ns	Not confirmed	Human rectal swab isolate, also mutation in other genes involved in colistin resistance	Dagher *et al*. (2020)
	ns	222	REC (1–112)	L216W	ns	Not confirmed	Rectal swab specimen, also mutation in other genes involved in colistin resistance	Dagher *et al*. (2020)
	ns	222	REC (1–112)	E217I	ns	Not confirmed	Rectal swab specimen, also mutation in other genes involved in colistin resistance	Dagher *et al*. (2020)

ST: sequence type; ns: not specified; confirmed (grey background): experimentally confirmed mutation mediating colistin resistance; not confirmed: mutation found by *in silico* analysis.

**Table 2.** Mutations in the histidine kinase PmrB of *Enterobacterales*.

Bacterial genera	ST	PmrB protein, length [aa]	Protein domain (residues)	Amino acid change	Resulting colistin MICs mg/L	Experimentally confirmed mutation	Bacterial source and comments	Reference
*E. coli*	648	363 (BasS)	–	Δ6-11 RPISLR	16	Confirmed	Human rectal swab isolate	Janssen *et al*.^2020^
	ns	363 (BasS)	–	Δ7–12	ns	Not confirmed	Human clinical specimen	Poirel, Jayol and Nordmann 2017
	59	363 (BasS)	–	L10P	4	Confirmed	Human urinary tract isolate	Cannatelli *et al*. 2017
	131	363 (BasS)	–	L10R	16	Confirmed	Human clinical blood isolate	Janssen *et al*. (2020)
	ns	363 (BasS)	–	L14Q	ns	Not confirmed	Avian pathogenic *E. coli*	Kathayat *et al*. (2020)
	38	363 (BasS)	TM1 (15–34)	G19E	4	Confirmed	Human rectal swab isolate	Janssen *et al*.^2020^
	131	363 (BasS)	TM1 (15–34)	Δ27-45 LISVFWLWHESTEQIQLFE	16	Confirmed	Human clinical specimen	Sato *et al*. (2018)
	ns	363 (BasS)	TM2 (66-88)	C84R or C84Y	ns	Not confirmed	Human clinical specimen	Bourrel *et al*. (2019)
	ns	363 (BasS)	HAMP (89–141)	T92P	ns	Not confirmed	Avian pathogenic *E. coli*	Kathayat *et al*.(2020)
	ns	363 (BasS)	HAMP (89–141)	R93P	ns	Not confirmed	Diseased pig	Kuang *et al*. 2020
	ns	363 (BasS)	HAMP (89–141)	P94A or P94L or P94Q or P94S	ns	Not confirmed	Human clinical specimen	Bourrel *et al*. (2019)
	10	363 (BasS)	HAMP (89–141)	A118T	>32	Not confirmed	Human clinical specimen	Luo *et al*. (2017)
	ns	363 (BasS)	HAMP (89–141)	E121K or E121Q	ns	Not confirmed	Human clinical specimen	Bourrel *et al*. (2019)
	14 /131	363 (BasS)	HAMP (89–141)	E123D	>32	Not confirmed	Human clinical specimen	Luo *et al*. (2017)
	ns	363 (BasS)	HAMP (89–141)	E123D	ns	Not confirmed	Diseased pig	Delannoy *et al*. (2017)
	ns	363 (BasS)	HisKA (142–202)	T147A	ns	Not confirmed	Human clinical specimen	Bourrel *et al*. (2019)
	ns	363 (BasS)	HisKA (142–202)	T156A	ns	Not confirmed	Diseased pig	Delannoy *et al*.(2017)
	ns	363 (BasS)	HisKA (142–202)	T156K	ns	Not confirmed	Human clinical specimen	Poirel *et al*. 2017
	ns	363 (BasS)	HisKA (142–202)	T156M	ns	Not confirmed	Human clinical specimen	Bourrel *et al*.(2019)
	ns	363 (BasS)	HisKA (142–202)	A159P	ns	Not confirmed	Human clinical specimen	Bourrel *et al*.(2019)
	ns	363 (BasS)	HisKA (142–202)	A159V	ns	Not confirmed	Human clinical specimen	Poirel *et al*. 2017
	ns	363 (BasS)	HisKA (142–202)	G160E	ns	Not confirmed	Diseased pig	Delannoy *et al*.(2017)
	ns	363 (BasS)	HisKA (142–202)	V161G	4	Not confirmed	Swine feces	Quesada *et al*.(2015)
	ns	363 (BasS)	HisKA (142–202)	E166K	ns	Not confirmed	Human clinical specimen	Bourrel *et al*.(2019)
	416/131	363 (BasS)	–	G206D	4/8	Confirmed	Human clinical specimen	Sato *et al*. (2018)
	641	363 (BasS)	HATPase_c (249–357)	D283G	8	Not confirmed	Calf ceacum, also mutation in PmrB Y358N	Rebelo *et al*.(2018)
	ns	363 (BasS)	HATPase_c (249–357)	D283G	ns	Not confirmed	Diseased pig	Delannoy *et al*. (2017)
	38	363 (BasS)	HATPase_c (249–357)	Y315F	>32	Not confirmed	Human clinical specimen	Luo *et al*.(2017)
	ns	363 (BasS)	HATPase_c (249–357)	V351I	ns	Not confirmed	Diseased pig	Delannoy *et al*. (2017)
	101/410	363 (BasS)	–	Y358N	>32	Not confirmed	Human clinical specimen	Luo *et al*. (2017)
	131	417 (BasS)	HAMP2	Extra HAMP domain	16	Confirmed	Human clinical blood isolate	Janssen *et al*. (2020)
*Klebsiella* spp.	ns	365	TM1 (13–35)	L17Q	32	Not confirmed	Human clinical specimen	Nordmann *et al*.(2016)
	17	365	TM1 (13–35)	G20S	256	Not confirmed	Chicken meat, also mutation in other genes involved in colistin resistance	Chaalal *et al*.(2021)
	258	365	–	V46E	ns	Not confirmed	Patient rectal swab	Gentile *et al*.(2020)
	646	365	–	Q56S	4	Not confirmed	Chicken meat, also mutation in other genes involved in colistin resistance	Chaalal *et al*. (2021)
	512	365	TM2 (67–89)	L82R	4	Confirmed	Human clinical specimen	Cannatelli *et al*.(2014)
	ns	365	TM2 (67–89)	S85R	ns	Not confirmed	Human feces from healthy individual	Olaitan *et al*.(2014)
	512	365	HAMP (90–142)	P95L	ns	Not confirmed	Human rectal swab isolate	Gentile *et al*.(2020)
	512	365	HAMP (90–142)	Δ129-134 ALNQLV	>8	Not confirmed	Human rectal swab isolate	Giordano *et al*. 2019
	ns	365	HAMP (90–142)	T140P	ns	Not confirmed	Human feces from healthy individuals	Olaitan *et al*. (2014)
	ns	365	HAMP (90–142)	H156R	ns	Not confirmed	Human clinical specimen	Macesic *et al*.(2020)
	14,258,15,101	365	HisKA (143–203)	T157P	3–6	Confirmed	Human clinical specimen	Jayol *et al*. 2014
	15	365	HisKA (143–203)	T157P	32	Not confirmed	Human clinical specimen	Cheng *et al*. (2015)
	86	365	–	T246A	64	Not confirmed	Human blood isolate, further mutations in other genes mediating colistin resistance	Cheong *et al*.(2020)
	11	365	HATPase_c (250–358)	R256G	>512	Not confirmed	Additional mutation in MgrB and PhoQ	Cheng *et al*. (2015)
	ns	365	HATPase_c (250–358)	H333Y	ns	Not confirmed	Human clinical specimen	Macesic *et al*. (2020)
	23	365	HATPase_c (250–358)	P344L	4	Not confirmed	Human blood isolate, further mutations in other genes mediating colistin resistance	Cheong *et al*.(2020)
*S. enterica*	45	365	TM1 (13–35)	Δ11–14	16	Confirmed	Human rectal swab	Olaitan *et al*. (2015)
*S*. Infantis	32	365	HAMP (89–141)	R92P	4 – >16	Not confirmed	Poultry farm	Jovcic *et al*. (2020)
	32	365	HisKA (142–202)	V164M or V164G	4 – >16	Not confirmed	Poultry farm	Jovcic *et al*. (2020)

ST: sequence type; ns: not specified; confirmed (grey background): experimentally confirmed mutation mediating colistin resistance; not confirmed: mutation found by *in silico* analysis; Δ: deletion.

*Escherichia coli* and *Salmonella* spp. exhibit most of the mutations that lead to colistin resistance in the genes of the PmrAB TCS, predominantly in the histidine-kinase PmrB. In contrast, fewer genetic alterations within PmrAB have been reported for klebsiellae.

Mutations in the transferase PmrC have been found in colistin-resistant *E. coli* and *Klebsiella* strains by *in silico* analysis (Table 3; Mathur *et al*. 2018, Choi *et al*. 2020). Notably, *pmrC* contains multiple missense mutations, but additional mutations in other colistin resistance-related genes have been found in the same isolates. Therefore, the contribution of gene alterations in *pmrC* to colistin resistance has not yet been deciphered.

**Table 3.** Mutations in the phosphoethanolamine transferase PmrC of *Enterobacterales*.

Bacterial genera	ST	PmrC protein, length [aa]	Protein domain (residues)	Amino acid change	Resulting colistin MICs mg/L	Experimentally confirmed mutation	Bacterial source and comments	Reference
*E. coli*	1	547	Transferase domain,-	T148A, K233T	8–16	Not confirmed	Isolate from animal and plant quarantine agency, also mutations in other genes mediating colistin resistance	Choi *et al*. (2020)
*Klebsiella* spp.	11, 14 and 231	546	TM1	C27F	4–16	Not confirmed	Human clinical specimen, also mutations in other genes mediating colistin resistance	Mathur *et al*. (2018)
		546	–	V39L				
		546	–	V42L				
		546	Transferase domain	R152H				
		546	Sulfatase domain	S260L				
		546	Sulfatase domain	S257L				
		546	Sulfatase domain	A279G				
		546	Sulfatase domain	Q319R				
		546	Sulfatase domain	D477N				

ST: sequence type; ns: not specified; confirmed (grey background): experimentally confirmed mutation mediating colistin resistance; not confirmed: mutation found by *in silico*.

### PhoPQ two-component system

The PhoQ sensor kinase has been shown to respond to low environmental Mg^2+^ concentrations, changes in pH and the presence of antimicrobial peptides resulting in activation and phosphorylation of the PhoP response regulator (Fig. 3). PhoP controls the expression of genes involved in magnesium transport and modification of LPS. Interestingly, PhoPQ was identified to regulate the expression of the small RNA *mgrR*, which is a negative regulator of the phosphoethanolamine transferase EptB (Moon and Gottesman, 2009). Additionally, PhoPQ contribute to colistin resistance by indirectly activating the PmrAB TCS via PmrD (Kox, Wosten and Groisman 2000; Kato, Latifi and Groisman 2003; Rubin *et al*. 2015). Missense mutations and deletions in PhoP have been identified in the REC and Trans_reg_C domains as well as inter-domain regions (Table 4; Cheng *et al*. 2015; Jayol *et al*. 2015; Delannoy *et al*. 2017; Dagher *et al*. 2020). Furthermore, mutations in PhoQ occurred in several functional domains, but also in inter-domain regions (Table 5; Choi and Ko 2014; Cheng *et al*. 2015; Olaitan *et al*. 2015; Halaby *et al*. 2016; Nordmann, Jayol and Poirel 2016; Luo *et al*. 2017; Gentile *et al*. 2020).

**Table 4.** Mutations in the response regulator PhoP of *Enterobacterales*.

Bacterial genera	ST	PhoP protein, length [aa]	Protein domain (residues)	Amino acid change	Resulting colistin MICs mg/L	Experimentally confirmed mutation	Bacterial source and comments	Reference
*E. coli*	ns	223	REC (1–112)	V108M	ns	not confirmed	Diseased pig	Delannoy *et al*. (2017)
	ns	223	Trans_reg_C (145–220)	A182P	ns	Not confirmed	Diseased pig	Delannoy *et al*. (2017)
*Klebsiella* spp.	29	223	REC (1–112)	V3F	>2048	Not confirmed	Human clinical specimen	Cheng *et al*. (2015)
	ns	223	REC (1–112)	L12Q	ns	Not confirmed	Human clinical specimen	Macesic *et al*. (2020)
	ns	223	REC (1–112)	L26Q	ns	Not confirmed	Human feces from healthy individuals	Olaitan *et al*. (2014)
	646	223	REC (1–112)	A30S	4	Not confirmed	Chicken meat, also mutation in other genes involved in colistin resistance	Chaalal *et al*. (2021)
	17	223	REC (1–112)	L87P	256	Not confirmed	Chicken meat, also mutation in other genes involved in colistin resistance	Chaalal *et al*. (2021)
	11	223	REC (1–112)	S86L	128	Not confirmed	Human clinical specimen	Cheng *et al*. (2015)
	ns	223	Trans_reg_C (145–220)	D191Y	12	Confirmed	Human clinical specimen	Jayol *et al*. (2015)
*Enterobacter* spp.	ns	223	REC (1–112)	D46V	ns	Not confirmed	Human rectal swab isolate, also mutation in other genes involved in colistin resistance	Dagher *et al*. (2020)
	ns	223	REC (1–112)	I47F	ns	Not confirmed	Human rectal swab isolate, also mutation in other genes involved in colistin resistance	Dagher *et al*. (2020)
	ns	223	REC (1–112)	I49F	ns	Not confirmed	Human rectal swab isolate, also mutation in other genes involved in colistin resistance	Dagher *et al*. (2020)
	ns	223	–	ΔE140	ns	Not confirmed	Human rectal swab isolate, also mutation in other genes involved in colistin resistance	Dagher *et al*. (2020)
	ns	223	–	ΔF141	ns	Not confirmed	Human rectal swab isolate, also mutation in other genes involved in colistin resistance	Dagher *et al*. (2020)
	ns	223	–	I143D	ns	Not confirmed	Human rectal swab isolate, also mutation in other genes involved in colistin resistance	Dagher *et al*. (2020)
	ns	223	–	N144A	ns	Not confirmed	Human rectal swab isolate, also mutation in other genes involved in colistin resistance	Dagher *et al*. (2020)
	ns	223	Trans_reg_C (145–220)	Δ148-163	ns	Not confirmed	Human rectal swab isolate, also mutation in other genes involved in colistin resistance	Dagher *et al*. (2020)

ST: sequence type; ns: not specified; confirmed (grey background): experimentally confirmed mutation mediating colistin resistance; not confirmed: mutation found by *in silico* analysis; Δ: deletion.

**Table 5.** Mutations in the histidine kinase PhoQ of *Enterobacterales*.

Bacterial genera	ST	PhoQ protein, length [aa]	Protein domain (residues)	Amino acid change	Resulting colistin MICs mg/L	Experimentally confirmed mutation	Bacterial source and comments	Reference
*E. coli*	354	486	–	N346K	>32	Not confirmed	Human clinical specimen	Luo *et al*. (2017)
	3997	486	HATPase_c (374–480)	E375K	4	Not confirmed	Human rectal swab isolate from healthy individuals	Olaitan *et al*. (2015)
*Klebsiella* spp.	ns	488	–	R16C	>128	Not confirmed	Human clinical specimen	Nordmann *et al*. (2016)
	43	488	TM1 (20-42)	A21S	16	Confirmed	Human clinical specimen	Halaby *et al*. (2016)
	ns	488	TM1 (20-42)	V24G	ns	Not confirmed	Human clinical specimen	Macesic *et al*. (2020)
	11	488	TM1 (20-42)	L26P	64	Not confirmed	Human clinical specimen	Cheng *et al*. (2015)
	ns	488	TM1 (20-42)	L30Q	ns	Not confirmed	Human clinical specimen	Macesic *et al*. (2020)
	ns	488	–	K46N	ns	Not confirmed	Human clinical specimen	Macesic *et al*. (2020)
	512	488	–	S56R	ns	Not confirmed	Human rectal swab isolate	Gentile *et al*. (2020)
	17	488	–	A70K, D90H	256	Not confirmed	Chicken meat, also mutation in other genes involved in colistin resistance	Chaalal *et al*. (2021)
	17	488	–	P72N, D90N	256	Not confirmed	Chicken meat, also mutation in other genes involved in colistin resistance	Chaalal *et al*. (2021)
	646	488	–	D74E, Q92S	4	Not confirmed	Chicken meat, also mutation in other genes involved in colistin resistance	Chaalal *et al*. (2021)
	646	488	–	I75L	4	Not confirmed	Chicken meat, also mutation in other genes involved in colistin resistance	Chaalal *et al*. (2021)
	944	488	–	E77D, K94E	4	Not confirmed	Chicken meat, also mutation in other genes involved in colistin resistance	Chaalal *et al*. (2021)
	258	488	–	L87P	ns	Not confirmed	Human rectal swab isolate	Gentile *et al*. (2020)
	ns	488	–	L96P	ns	Not confirmed	Human clinical specimen	Olaitan *et al*. (2014)
	ns	488	–	I109N	ns	Not confirmed	Human clinical specimen	Macesic *et al*. (2020)
	11	488	–	D150G	128	Not confirmed	Human clinical specimen, additional mutation in MgrB and PmrB R156G	Cheng *et al*. (2015)
	ns	488	–	S174N	4	Not confirmed	Human clinical specimen	Choi and Ko (2014)
	ns	488	TM2 (194-216)	P208H	ns	Not confirmed	Human clinical specimen	Macesic *et al*. (2020)
	15	488	HAMP (215-266)	V258F	64	Not confirmed	Human clinical specimen, additional mutation in MgrB	Cheng *et al*. (2015)
	ns	488	HisKA (274-482)	Q310L	ns	Not confirmed	Human clinical specimen	Macesic *et al*. (2020)
	ns	488	HisKA (274-482)	H339D	ns	Not confirmed	Human clinical specimen	Macesic *et al*. (2020)
	ns	488	HisKA (274-482)	L348Q	ns	Not confirmed	Human clinical specimen	Olaitan *et al*. (2014)
	ns	488	HisKA (274-482)	A351P	ns	Not confirmed	Human clinical specimen	Macesic *et al*. (2020)
	ns	488	HisKA (274-482)	G385S	ns	Not confirmed	Human clinical specimen	Macesic *et al*. (2020)
	ns	488	HisKA (274-482)	P420S	ns	Not confirmed	Human clinical specimen	Macesic *et al*. (2020)
	512	489 (insertion)		D266_267insD	ns	Not confirmed	Human rectal swab isolate, ins799/801(GAC)	Gentile *et al*. (2020)

ST: sequence type; ns: not specified; confirmed (grey background): experimentally confirmed mutation mediating colistin resistance; not confirmed: mutation found by *in silico* analysis

### PmrD adaptor protein

PmrD is a small protein connecting the two TCS's PmrAB and PhoPQ (Fig. 3). PmrD binds to the phosphorylated form of PmrA following activation by PhoP (Kato and Groisman, 2004; Mitrophanov *et al*. 2008; Rubin *et al*. 2015). Phosphorylated and therefore activated PmrA is protected from dephosphorylation resulting in binding to its targets such as the promotor of the *pmrHFIJKLM* operon, which encodes genes responsible for the modification of LPS. Interestingly, gene multiplications of *pmrD* positively correlate with colistin resistance levels in *Salmonella*, but not in *E. coli* (Hjort, Nicoloff and Andersson 2016). Furthermore, *pmrD* is not present in all *Enterobacter* species (Guerin *et al*. 2016).

Missense mutations in PmrD in colistin-resistant *Enterobacterales* have been found in human clinical *Klebsiella pneumoniae* (*K. pneumoniae*) isolates and *E. coli* strains of animal origin (Table 6; Kim *et al*. 2019; Cheong *et al*. 2020). However, the parallel occurrence of mutations in MgrB, PhoPQ or PmrAB does not clarify the contribution of mutations in PmrD to the colistin resistance phenotype. Notably, a missense mutation at position K82 has been identified in several *E. coli* strains and represents eventually a hot spot (Kim *et al*. 2019). Furthermore, this stand-alone mutation occurred in strains without other detectable genetic alterations eventually highlighting this aa substitution as critical for colistin resistance. Nevertheless, experimental confirmation using site-directed mutagenesis or complementation experiments is required.

**Table 6.** Mutations in the connector protein PmrD of *Enterobacterales*.

Bacterial genera	ST	PmrD protein, length [aa]	Protein domain (residues)	Amino acid change	Resulting colistin MICs mg/L	Experimentally confirmed mutation	Bacterial source and comments	Reference
*E. coli*	ns	88	–	N11D	ns	Not confirmed	Animal samples, also mutations in other genes involved in colistin resistance	Kim *et al*. (2019)
	ns	88	–	M20	ns	Not confirmed	Animal samples, also mutations in other genes involved in colistin resistance	Kim *et al*. (2019)
	ns	88	–	A27T	ns	Not confirmed	Animal samples, also mutations in other genes involved in colistin resistance	Kim *et al*. (2019)
	ns	88	–	K35N	ns	Not confirmed	Animal samples, also mutations in other genes involved in colistin resistance	Kim *et al*. (2019)
	ns	88	–	A52V	ns	Not confirmed	Animal samples, also mutations in other genes involved in colistin resistance	Kim *et al*. (2019)
	3054, 224, 6488, 2035, 278, 448, 906, 4038, 156 and 548	88	–	K82T	4-32	Not confirmed	Animal sample	Kim *et al*. (2019)
*Klebsiella* spp.	ns	81	–	Q9R	ns	Not confirmed	Human blood isolate, also mutation in other genes involved in colistin resistance	Cheong *et al*. (2020)
	ns	81	–	A12S	ns	Not confirmed	Human blood isolate, also mutation in other genes involved in colistin resistance	Cheong *et al*. (2020)
	ns	81	–	S13M	ns	Not confirmed	Human blood isolate, also mutation in other genes involved in colistin resistance	Cheong *et al*. (2020)
	ns	81	–	A14T	ns	Not confirmed	Human blood isolate, also mutation in other genes involved in colistin resistance	Cheong *et al*. (2020)
	ns	81	–	L16S	ns	Not confirmed	Human blood isolate, also mutation in other genes involved in colistin resistance	Cheong *et al*. (2020)
	ns	81	–	R18C	ns	Not confirmed	Human blood isolate, also mutation in other genes involved in colistin resistance	Cheong *et al*. (2020)
	ns	81	–	A25T	ns	Not confirmed	Human blood isolate, also mutation in other genes involved in colistin resistance	Cheong *et al*. (2020)
	ns	81	–	E27A	ns	Not confirmed	Human blood isolate, also mutation in other genes involved in colistin resistance	Cheong *et al*. (2020)
	ns	81	–	R38H	ns	Not confirmed	Human blood isolate, also mutation in other genes involved in colistin resistance	Cheong *et al*. (2020)
	ns	81	–	R40Q	ns	Not confirmed	Human blood isolate, also mutation in other genes involved in colistin resistance	Cheong *et al*. (2020)
	ns	81	–	D50N	ns	Not confirmed	Human blood isolate, also mutation in other genes involved in colistin resistance	Cheong *et al*. (2020)
	ns	81	–	T60A	ns	Not confirmed	Human blood isolate, also mutation in other genes involved in colistin resistance	Cheong *et al*. (2020)
	ns	81	–	R66L	ns	Not confirmed	Human blood isolate, also mutation in other genes involved in colistin resistance	Cheong *et al*. (2020)
	ns	81	–	N67K	ns	Not confirmed	Human blood isolate, also mutation in other genes involved in colistin resistance	Cheong *et al*. (2020)
	ns	81	–	T77A	ns	Not confirmed	Human blood isolate, also mutation in other genes involved in colistin resistance	Cheong *et al*. (2020)
	ns	81	–	N78K	ns	Not confirmed	Human blood isolate, also mutation in other genes involved in colistin resistance	Cheong *et al*. (2020)
	ns	81	–	A79L	ns	Not confirmed	Human blood isolate, also mutation in other genes involved in colistin resistance	Cheong *et al*. (2020)
	ns	81	–	G80D	ns	Not confirmed	Human blood isolate, also mutation in other genes involved in colistin resistance	Cheong *et al*. (2020)
	ns	81	–	K81G	ns	Not confirmed	Human blood isolate, also mutation in other genes involved in colistin resistance	Cheong *et al*. (2020)

ST: sequence type; ns: not specified; confirmed (grey background): experimentally confirmed mutation mediating colistin resistance; not confirmed: mutation found by *in silico* analysis

### MgrB regulator

MgrB is a 47 aa regulatory transmembrane peptide, which is produced upon activation of the PhoPQ system (Lippa and Goulian, 2009). Through interactions with the periplasmic domain of PhoQ, MgrB acts as a feedback inhibitor of the PhoPQ system in different bacteria (Fig. 3). Within *mgrB* various groups of genetic changes have been identified including missense mutations, non-sense mutations, deletion of individual nucleotides or deletion of the entire *mgrB* locus as well as insertions of additional aas and IS elements (Table 7; Cannatelli *et al*. 2014; Olaitan *et al*. 2014; Cheng *et al*. 2015; Nordmann, Jayol and Poirel 2016; Haeili *et al*. 2017; Esposito *et al*. 2018). Almost any position of the *mgrB* gene can be affected, which predominantly leads to functional inactivation of the peptide. Thus, the PhoPQ system becomes upregulated, which, in turn, activates the Pmr system responsible for modification of the LPS. In colistin-resistant *K. pneumoniae* strains, the disruption of the *mgrB* gene plays a significant role, with a prevalence of up to 59% of human clinical isolates tested (Cannatelli *et al*. 2014). A study analyzing 973 clinical *K. pneumoniae* isolates showed that the insertions of IS elements (IS5-like, IS1F, ISKpn13, ISKpn14 and IS10R) are the most common genetic alteration of *mgrB*, followed by partial or complete deletion of the gene, missense mutations and finally nonsense mutations (Hamel *et al*. 2020). It seems that the high frequency of mutational changes and the inactivation of MgrB do not have major detectable consequences for the fitness and virulence of the *K. pneumoniae* strains. In contrast to *K. pneumoniae*, only two missense mutations in MgrB of *E. coli* have been reported, but their contribution to colistin resistance has not yet been confirmed, since no complementation or site-directed mutagenesis has been performed (Delannoy *et al*. 2017). Noteworthy, the *phoPQ* operon in *E. coli* is regulated not only by MgrB but also by the small RNA MicA, which eventually negates the contribution of mutational changes in MgrB to colistin resistance (Janssen *et al*. 2020). In *Enterobacter* a few mutations in MgrB have been identified by *in silico* analysis, however, concurrent mutations in PmrAB or PhoP of the same isolate were present, making it difficult to assess their significance for colistin resistance (Dagher *et al*. 2020).

**Table 7.** Mutations in the regulator MgrB of *Enterobacterales*.

Bacterial genera	ST	MgrB protein, length [aa]	Protein domain (residues)	Amino acid change	Resulting colistin MICs mg/L	Experimentally confirmed mutation	Bacterial source and comments	Reference
*E. coli*	ns	47	–	V8A	ns	Not confirmed	Diseased pig	Delannoy *et al*. (2017)
	ns	47	–	Q33R	ns	Not confirmed	Diseased pig	Delannoy *et al*. (2017)
*Klebsiella* spp.	ns	47	–	K3*	64–128	Not confirmed	Human clinical specimen	Nordmann *et al*. (2016)
	ns	47	–	L9*	8–12	Not confirmed	Human feces from healthy individuals	Olaitan *et al*. (2014)
	ns	47	–	I13*	16–32	Not confirmed	Human feces from healthy individuals	Olaitan *et al*. (2014)
	491	47	–	A14S	12–4	Not confirmed	Human feces from healthy individuals	Olaitan *et al*. (2014)
	726	47	–	C16*	>128	Not confirmed	Healthy broiler, also mutation in PmrB R256G and CrrB T150R	Pishnian *et al*. (2019)
	101	47	–	L17R	32	Confirmed	Human blood isolate	Esposito *et al*. (2018)
	ns	47	–	W20R	32	Not confirmed	Human clinical specimen	Nordmann *et al*. (2016)
	258	47	–	L24H	32	Confirmed	Human clinical specimen	Cannatelli *et al*. (2014)
	507	47	–	V26*	32	Not confirmed	Human feces from healthy individuals	Olaitan *et al*. (2014)
	ns	47	–	M27K	32	Not confirmed	Human clinical specimen	Nordmann *et al*. (2016)
	1309	47	–	C28F	4	Not confirmed	Human feces from healthy individual	Olaitan *et al*. (2014)
	258	47	–	C28Y	32	Confirmed	Human clinical specimen	Cannatelli *et al*. (2014)
	ns	47	–	C28*	32– >128	Not confirmed	Human clinical specimen	Nordmann *et al*. (2016)
	ns	47	–	Q30*	32–128	Confirmed	Human clinical specimen	Nordmann *et al*. (2016); Haeili *et al*. (2017)
	ns	47	–	D31N	ns	Not confirmed	Human clinical specimen	Olaitan *et al*. (2014)
	101	47	–	V32G	2–56	Confirmed	Human blood isolate	Esposito *et al*. (2018)
	ns	47	–	Q33*	32	Not confirmed	Human clinical specimen	Nordmann *et al*. (2016)
	ns	47	–	F35I	ns	Not confirmed	Human feces from healthy individual	Olaitan *et al*. (2014)
	258/512	47	–	G37S	16–32	Confirmed	Human clinical specimen	Cannatelli *et al*. (2014)
	ns	47	–	C39Y	64	Not confirmed	Human clinical specimen	Nordmann *et al*. (2016)
	ns	47	–	C39*	128	Confirmed	Human clinical specimen	Haeili *et al*. (2017)
	ns	47	–	N42Y, K43I	32	Not confirmed	Human clinical specimen	Nordmann *et al*. (2016)
	ns	47	–	I45T	64	Not confirmed	Human clinical specimen	Nordmann *et al*. (2016)
	ns	47	–	P46S	64	Not confirmed	Human clinical specimen	Nordmann *et al*. (2016)
	ns	47	–	W47R or W47*	4 or 32	Not confirmed	Human clinical specimen	Nordmann *et al*. (2016)
	ns	62	–	*48Y	ns	Confirmed	Human clinical specimen	Cheng *et al*. (2015)
	ns	Additional insertions	–	V7::ISEcp1/blaCTX-M-15 or V7::IS1R	64	Not confirmed	Human clinical specimen	Nordmann *et al*. (2016)
	ns	Additional insertions	–	V12::IS102-like	32	Not confirmed	Human clinical specimen	Nordmann *et al*. (2016)
	ns	Additional insertions	–	L14::IS102-like orL14::IS903b orL14::IS2 orL14::IS1R	>1286 464128	Not confirmed	Human clinical specimen	Nordmann *et al*. (2016)
	ns	Additional insertions	–	W20::IS1R	64	Not confirmed	Human clinical specimen	Nordmann *et al*. (2016)
	ns	Additional insertions	–	V23::IS903b-like or V23::IS903-like	64 or 128	Not confirmed	Human clinical specimen	Nordmann *et al*. (2016)
	ns	Additional insertions	–	F24::IS5-like orF24::ISKpn13 orF24::ISKpn26-like	16–12812864– >128	Not confirmed	Human clinical specimen	Nordmann *et al*. (2016)
	ns	Additional insertions	–	N25::SKpn26-like orN25::IS903B orN25::ISKpn14 or	3232128	Not confirmed	Human clinical specimen	Nordmann *et al*. (2016)
	258/512	Additional insertions	–	N25::IS5-like element	16–32 / 16–32	Confirmed	Human clinical specimen	Cannatelli *et al*. (2014)
	ns	Additional insertions	–	D31::IS903b	64	Not confirmed	Human clinical specimen	Nordmann *et al*. (2016)
	512	Additional insertions	–	F35::IS1F-like element	8-32	Confirmed	Human clinical specimen	Cannatelli *et al*. (2014)
	ns	Additional insertions	–	I38::IS1R-like orI38::IS903b-like	12 864	Not confirmed	Human clinical specimen	Nordmann *et al*. (2016)
	ns	Additional insertions	–	C39::IS1R	8	Not confirmed	Human clinical specimen	Nordmann *et al*. (2016)
	ns	Additional insertions	–	I41::ISKpn26-like orI41::IS1R	12 832	Not confirmed	Human clinical specimen	Nordmann *et al*. (2016)
	147	Additional insertions	–	I41::ISKpn14	8–16	Confirmed	Human clinical specimen	Cannatelli *et al*. (2014)
	ns	Additional insertions	–	N42::ISKpn14	64	Not confirmed	Human clinical specimen	Nordmann *et al*. (2016)
	512	Additional insertions	–	N42::IS5-like element	32	Confirmed	Human clinical specimen	Cannatelli *et al*. (2014)
	ns	Additional insertions	–	Q43::IS1R	128	Not confirmed	Human clinical specimen	Nordmann *et al*. (2016)
	ns	Insertions upstream of mgrB	–	nt -62 to -26	32–128	Not confirmed	Human clinical specimen	Nordmann, Jayol and Poirel (2016)
	512	Deletions	–	Δnt18/27	32	Confirmed	Human clinical specimen, frameshift and premature termination	Cannatelli *et al*. (2014)
	512	Deletions	–	Δnt19	128–256	Confirmed	Human blood isolate, frameshift and premature termination	Esposito *et al*. (2018)
	258/512	Deletions	–	ΔmgrB locus	64 /8	Confirmed	Human clinical specimen	Cannatelli *et al*. (2014)
	258	Deletions	–	ΔmgrB locus	16	Confirmed	Human clinical specimen, deletion from −400 to +599	Cannatelli *et al*. (2014)
	512	Deletions	–	Δnt61/70	ns	Not confirmed	Human rectal swab isolate	Gentile *et al*. (2020)
	258/512	Deletions	–	Δnt47	32 / 8	Confirmed	Human clinical specimen, frameshift and premature termination	Cannatelli *et al*. (2014)
	512	Deletions	–	Δnt 109/119	32	Confirmed	Frameshift and premature termination	Cannatelli *et al*. (2014)
	258	47 (non-sense mutation)	–	c88t	64	Confirmed	Human blood isolate, non-sense mutation and premature termination	Esposito *et al*. (2018)
*Enterobacter* spp.	ns	47	–	V38S or V38I	ns	Not confirmed	Human rectal swab isolate, also mutation in other genes involved in colistin resistance	Dagher *et al*. (2020)
	ns	47	–	C39G	ns	Not confirmed	Human rectal swab isolate, also mutation in other genes involved in colistin resistance	Dagher *et al*. (2020)
	ns	47	–	A40K	ns	Not confirmed	Human rectal swab isolate, also mutation in other genes involved in colistin resistance	Dagher *et al*. (2020)
	ns	47	–	I41M	ns	Not confirmed	Human rectal swab isolate, also mutation in other genes involved in colistin resistance	Dagher *et al*.(2020)
	ns	47	–	N42S	ns	Not confirmed	Human rectal swab isolate, also mutation in other genes involved in colistin resistance	Dagher *et al*. (2020)
	ns	47	–	K43G	ns	Not confirmed	Human rectal swab isolate, also mutation in other genes involved in colistin resistance	Dagher *et al*. (2020)
	ns	47	–	I45Y	ns	Not confirmed	Human rectal swab isolate, also mutation in other genes involved in colistin resistance	Dagher *et al*. (2020)
	ns	47	–	P46G	ns	Not confirmed	Human rectal swab isolate, also mutation in other genes involved in colistin resistance	Dagher *et al*. (2020)
	ns	47	–	W47V or W47S	ns	Not confirmed	Human rectal swab isolate, also mutation in other genes involved in colistin resistance	Dagher *et al*. (2020)

ST: sequence type; ns: not specified; confirmed (grey background): experimentally confirmed mutation mediating colistin resistance; not confirmed: mutation found by *in silico* analysis; *, stop codon results in termination and truncated protein; Δ, deletion; ::, insertion

### CrrAB two-component system

CrrA and CrrB belong to a third TCS, which has been investigated in the context of colistin resistance in *K. pneumoniae*. The physiologic role of the TCS is still unknown. The *crrAB* operon is variably expressed in *K. pneumonia*e and has also been found in *Enterobacter* spp., but is not encoded in the *E. coli* chromosome (Wright *et al*. 2015). CrrB mutations have been reported to increase CrrC expression, which positively regulates the PmrAB TCS, thereby resulting in elevated transcription of the *pmrC* gene and the *pmrHFIJKLM* operon (Fig. 3; Cheng *et al*. 2016). The *pmrHFIJKLM* operon can also be directly activated by the CrrAB TCS, which has been shown by using CrrB mutants in *K. pneumoniae* (McConville *et al*. 2020). A single missense mutation has been identified in the response regulator CrrA of *K. pneumoniae*, whereas several confirmed missense mutations in the histidine kinase CrrB were found to induce colistin resistance (Table 8; Wright *et al*. 2015; Cheng *et al*. 2016; Jayol *et al*. 2017; Pishnian, Haeili and Feizi 2019).

**Table 8.** Mutations in the CcrAB two-component system of *Enterobacterales*.

Bacterial genera	ST	Protein, length [aa]	Protein domain (residues)	Amino acid change	Resulting colistin MICs mg/L	Experimentally confirmed mutation	Bacterial source and comments	Reference
*Klebsiella* spp.	11	CrrA (234)		A83V	>128	Not confirmed	Dead broiler, also mutation in PmrB R256G	Pishnian, Haeili and Feizi (2019)
*Klebsiella* spp.	258a	CrrB (353)	–	Q10L	16	Confirmed	Human clinical isolate	Wright *et al*. (2015)
	ns	CrrB (353)	TM1 (12–34)	Y31H	512	Confirmed	Human clinical isolate	Cheng *et al*. (2016)
	ns	CrrB (353)	HAMP (81–135)	F84S	>128	Confirmed	Human clinical isolate	Jayol *et al*. (2017)
	ns	CrrB (353)	HAMP (81–135)	L87V	ns	Not confirmed	Human clinical specimen	Macesic *et al*. (2020)
	258a	CrrB (353)	HAMP (81–135)	L94M	16	Confirmed	Human clinical isolate	Wright *et al*. (2015)
	ns	CrrB (353)	HisKA (136–200)	W140R	2048	Confirmed	Human clinical isolate	Cheng *et al*. (2016)
	ns	CrrB (353)	HisKA (136–200)	N141I or N141Y	2048 / >128	Confirmed	Human clinical isolate	Cheng *et al*. (2016), Jayol *et al*. (2017)
	ns	CrrB (353)	HisKA (136–200)	P151S or P151L	1024 / >128	Confirmed	Human clinical isolate	Cheng *et al*. (2016); Jayol *et al*. (2017)
	ns	CrrB (353)	HisKA (136–200)	G183V	>128	Confirmed	Human clinical isolate	Jayol *et al*. (2017)
	ns	CrrB (353)	HisKA (136–200)	L191F	ns	Not confirmed	Human clinical specimen	Macesic *et al*. (2020)
	ns	CrrB (353)	HisKA (136–200)	S195N	2048	Confirmed	Human clinical isolate	Cheng *et al*. (2016)
	ns	CrrB (353)	–	S322W	ns	Not confirmed	Human clinical specimen	Macesic *et al*. (2020)
*C. freundii*	117	CrrB (353)	HAMP (81–135)	A91T	256	Not confirmed	Human clinical specimen	Rocha *et al*. (2020)

ST: sequence type; ns: not specified; confirmed (grey background): experimentally confirmed mutation mediating colistin resistance; not confirmed: mutation found by *in silico* analysis

### The alterated proteins YciM, LpxM, RamA and OmpW of *K. pneumoniae*

In *K. pneumoniae*, additional genes seem to be associated with the colistin-resistant phenotype. YciM (LapB in *E. coli*) has not been well-characterized in *K. pneumoniae*, however, its homolog in *E. coli* is involved in maintaining the cell wall integrity by regulation of LPS biosynthesis (Mahalakshmi *et al*. 2014; Nicolaes *et al*. 2014). A total of two missense mutations in YciM have been detected in human clinical isolates of which the mutation V43G has been experimentally confirmed to cause colistin resistance (Table 9; Halaby *et al*. 2016; Boszczowski *et al*. 2019). LpxM (MsbB) is responsible for the acylation of Lipid A in *Enterobacterales* (Somerville *et al*. 1996; Khan *et al*. 1998). Interestingly, the loss of LpxM leads to increased colistin susceptibility (Clements *et al*. 2007). Four mutations have been found in *K. pneumoniae*, but only the mutation V30G was confirmed to contribute to colistin resistance (Halaby *et al*. 2016; Boszczowski *et al*. 2019). Very recently, a single missense mutation has been described in the global regulator RamA of a colistin-resistant human clinical *K. pneumoniae* isolate (Table 9; Macesic *et al*. 2020). RamA is responsible for the activation of gene expression necessary for the biosynthesis and modification of lipid A (De Majumdar *et al*. 2015). Furthermore, the same study detected an IS-insertion in the outer membrane protein OmpW, likely leading to its functional inactivation, in a clinical *K. pneumoniae* strain resistant to colistin (Macesic *et al*. 2020). This is the first indication of the involvement of OmpW in the colistin-resistant phenotype of *Enterobacterales*. In contrast, OmpW of *A. baumannii*, which is a homolog to OmpW of *E. coli*, showed reduced expression levels in colistin-resistant strains and was involved in binding to colistin (Vila, Marti and Sanchez-Cespedes 2007; Catel-Ferreira *et al*. 2016).

**Table 9.** Additional colistin resistance-associated genes in *Enterobacterales*

Bacterial genera	ST	Protein, length [aa]	Protein domain (residues)	Amino acid change	Resulting colistin MICs mg/L	Experimentally confirmed mutation	Bacterial source and comments	Reference
*Klebsiella* spp.	43	YciM (LapB) (389)	–	V43G	48	Confirmed	Human clinical isolate	Halaby *et al*. (2016)
	11	YciM (LapB) (389)	–	N212T	ns	Not confirmed	Human clinical isolate, also mutation in other genes involved in colistin resistance	Boszczowski *et al*. (2019)
*Klebsiella* spp.	11	LpxM (324)	–	N6K	ns	Not confirmed	Human clinical isolate, also mutation in other genes involved in colistin resistance	Boszczowski *et al*. (2019)
	43	LpxM (324)	–	V30G	8	Confirmed	Human clinical isolate	Halaby *et al*. (2016)
	11, 23 340	LpxM (324)	–	S285G	ns	Not confirmed	Human clinical isolate, also mutation in other genes involved in colistin resistance	Boszczowski *et al*. (2019)
	11	LpxM (324)	–	P321T	ns	Not confirmed	Human clinical isolate, also mutation in other genes involved in colistin resistance	Boszczowski *et al*. (2019)
*Klebsiella* spp.	ns	RamA		V82A	ns	Not confirmed	Human clinical isolate	Macesic *et al*. (2020)

ST: sequence type; ns: not specified; confirmed (grey background): experimentally confirmed mutation mediating colistin resistance; not confirmed: mutation found by *in silico* analysis.

There are excellent publications on the mechanisms of polymyxin resistance and the importance of chromosomal mutations, but these studies also include *in vitro* induced mutations or mention only a portion of all the so far identified genes. We provide here a summary of fourteen chromosomally encoded genes that have been analyzed in the context of colistin resistance in *Enterobacterales* of animal and human origin. However, five genes (*prmC*, *pmrD*, *crrA*, *ramA* and *ompW*) were not experimentally confirmed to mediate colistin resistance and additional mutations in other colistin resistance genes were found in the same isolate. The cumulative appearance of genetic alterations in different colistin resistance genes seems to be a common phenomenon. Interestingly, Macesic *et al*. (2020) found a positive correlation between the number of mutations and the MIC value of a colistin-resistant isolate. Overall, the majority of suggested missense mutations in those fourteen genes were found by *in silico* analysis and not experimentally validated, which complicates the identification of genes and associated mutations crucial for the colistin resistance phenotype in *Enterobacterales*. Therefore, the summarized mutations (Tables 1–9) are likely to be an overestimation of the actual number of genetic changes that cause resistance to colistin. Furthermore, many studies analyze the sequence of only a limited number of genes, e.g. only *pmrAB*, and may not identify the critical mutation(s) responsible for colistin resistance. Vice versa, genetic changes are also found in colistin resistance determinants of susceptible isolates, which complicates the identification of causative mutations in resistant strains. Most likely, not all genes that play a role in the colistin-resistant phenotype have been identified so far. Interestingly, *Enterobacterales* isolates have also been found to have a dual resistance mechanism as they harbor a plasmid-borne *mcr*-gene and mutational alterations in colistin resistance genes (Garcia-Menino *et al*. 2019; Zakaria, Edward and Mohamed 2021). Those observations raise new questions that need to be addressed: Which mechanism was developed first and did it provide sufficient resistance? Is there an additive effect of both resistance mechanism? Did one mechanism even facilitate the acquisition of the second mechanism? Is the presence of the *mcr-*gene only accidental, since other beneficial genes are encoded on the plasmid?

### Species-specific colistin resistance mechanisms

Especially in *K. pneumoniae*, two more resistance mechanisms have been described which are not based on mutational changes in chromosomal genes. The overproduction and shedding of anionic capsular polysaccharide prevents cationic polymyxins to reach their target on the outer membrane (Llobet, Tomas and Bengoechea 2008). In addition, the overexpression of efflux pumps, such as AcrAB and KpnEF, has been suggested as an effective mechanism to exfiltrate the antibiotic from the bacterial cell (Padilla *et al*. 2010; Srinivasan and Rajamohan, 2013; Naha *et al*. 2020).

### Colistin and the polymyxin family

The family of polymyxins comprises five antimicrobial compounds (polymyxin A, B, C, D and E). Due to their reduced renal toxicity compared to the other polymyxins, only polymyxin B and E (colistin) are used as last-resort defense against severe infections with CRE in human medicine (Li, Nation and Kaye 2019). The polymyxins share a similar structure and are pentacationic polypeptides consisting of a cyclic heptapeptide linked to a linear tripeptide, whose N-terminus is acylated with a fatty acid moiety. Colistin is a secondary metabolite peptide, which is nonribosomal produced by the soil bacterium *Paenibacillus polymyxa* (formerly named *Bacillus polymyxa*). Since its introduction in the 1950s, colistin has been used continuously in the veterinary medicine to treat and prevent animal infectious diseases caused by Gram-negative bacteria. For the treatment of human infections, colistin was initially used therapeutically in Japan and in Europe during the 1950s and in the United States in 1959. However, the intravenous formulations of colistin and polymyxin B were gradually abandoned in most parts of the world in the early 1980s. Colistin was restricted to ophthalmic and topical use owing to concerns about neurotoxicity and nephrotoxicity (Ryan *et al*. 1969; Brown, Dorman and Roy 1970; Koch-Weser *et al*. 1970). Thereafter, colistin was re-introduced for systemic treatment of lung infections due to MDR, Gram-negative bacteria in patients with cystic fibrosis (Conway *et al*. 1997; Ledson *et al*. 1998). Given the increased detection of colistin-resistant bacteria in livestock animals and animal-related food products as well as the need to retain the efficacy of antimicrobials to treat MDR infections in humans, the use of colistin in veterinary medicine is being re-evaluated. Throughout this review, and unless otherwise indicated, the term polymyxins is used to refer to the two clinically relevant compounds, polymyxin B and colistin.

## COLISTIN USAGE IN EUROPE

### Colistin resistance in the food, animal and livestock sector

Colistin is mainly administered orally in form of premix, powder and oral solutions in feed, drinking water or during milk replacer diets for the treatment of gastrointestinal tract infections caused by non-invasive *E. coli*. Colistin products are given to an enormous amount of different animal species including pigs, poultry, cattle, sheep, goats, laying hens and rabbits, but also to milk-producing species such as cattle, sheep and goats. Within the European Union (EU) and the European Economic Area (EEA), colistin and polymyxin B are authorized at national level and have been used since the 1950s (Table S2, Supporting Information). Colistin was widely used among the European countries in food-producing animals and their consumption extended beyond the treatment of infections to include pro- and meta-phylaxis purposes. At that time, primary indications were the treatment and prevention of diarrhoea in pigs caused by *E. coli* and *Salmonella* spp., the treatment of neonatal diarrhoea in piglets and veal calves caused by *E. coli*, as well as the treatment of mild colibacillosis in poultry (Timmerman *et al*. 2006; Pardon *et al*. 2012).

However, the extensive use of colistin has led to the emergence and spread of AMR pathogenic and commensal bacteria in the intestinal tract of food-producing animals. Resistant bacteria could colonize the human microbiota via the food chain through handling and/or consumption of contaminated food products. In light of this, initial restrictions on the use of colistin were implemented in the EU in 2006, prohibiting the supplementation of animal feed with antibiotics to promote animal growth (Regulation 1831/2003/EC; European Commission 2005). Importantly, in 2014 the EU implemented mandatory susceptibility testing to colistin for bacteria isolated from food-producing animals covered by the national monitoring programs (Regulation 2013/652/EU; European Commission 2013).

**Figure 1.**
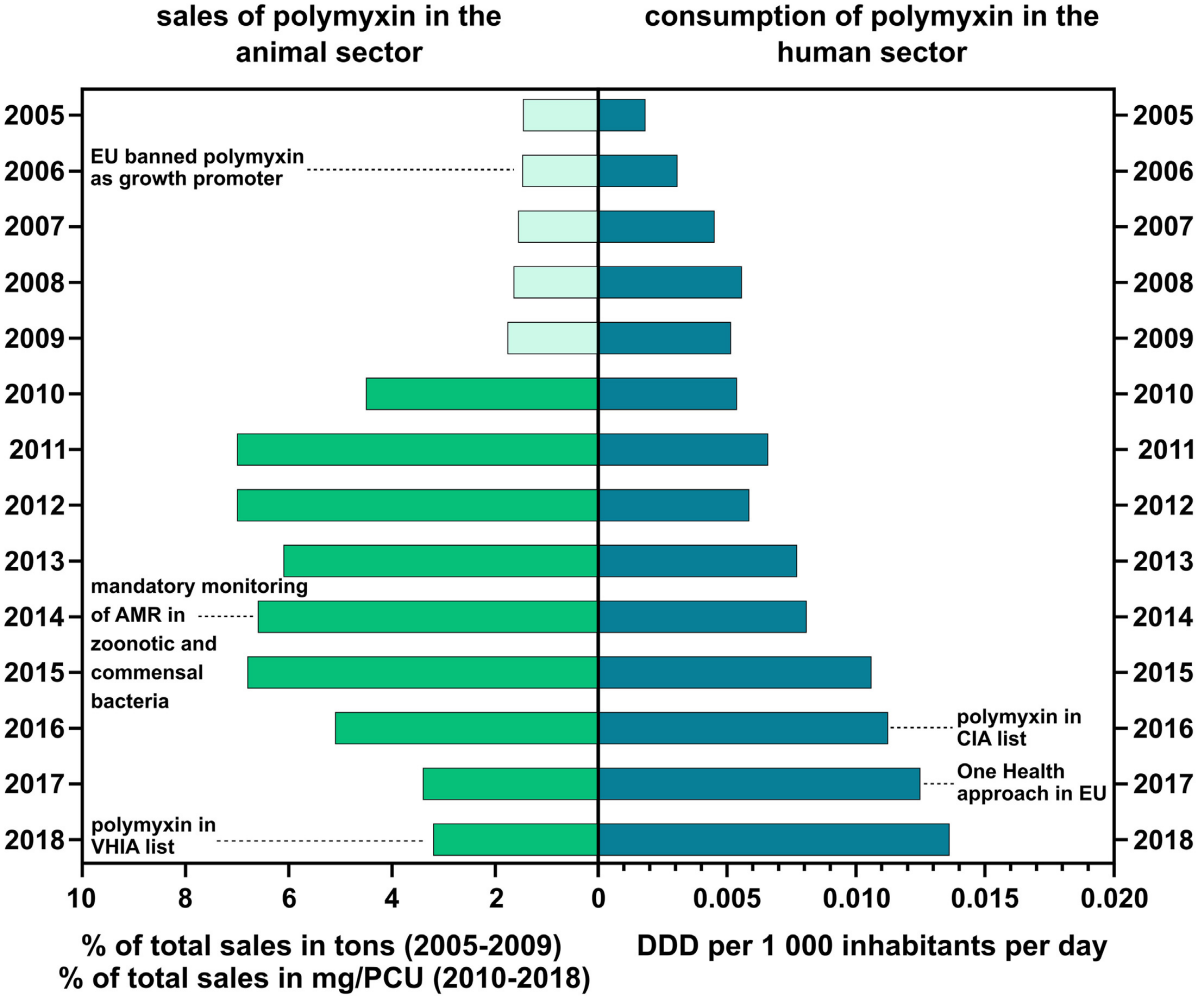
Comparison of changes in sales and consumption of polymyxin in veterinary and human medicine within the European Union. Left: Averaged percentages of sales of polymyxin relative to total sales (in tons of active ingredient) in five European countries for the period 2005–2009 using the data from the first ESVAC report (European Medicines Agency 2011). The period 2010–2017 shows the averaged percentage of sales of polymyxin relative to the total sales (in mg of active ingredient by Population Correction Unit (PCU)) of the reporting countries for each year (European Medicines Agency 2012; European Medicines Agency 2013; European Medicines Agency 2014; European Medicines Agency 2015; European Medicines Agency 2016; European Medicines Agency 2017; European Medicines Agency 2018; European Medicines Agency 2019; European Medicines Agency 2020). Right: Annual average consumption of polymyxin [in Defined Daily Doses (DDD) per 1000 inhabitants per day] in the community and hospital sector in Europe, including Switzerland, using data provided by the ESAC-Net interactive database (https://www.ecdc.europa.eu/en/antimicrobial-consumption/surveillance-and-disease-data/database, accessed October 2020). For both sectors, polymyxins include colistin (polymyxin E) and polymyxin B.

In 2011, the sales of antimicrobials were collected on EU level and published standardized and corrected for the total weight of treated animals. Following the reports of 25 EU/EEA members, polymyxin was the 5th most sold class of antimicrobials after tetracyclines (37%), penicillins (23%), sulphonamides (11%) and macrolides (8%) (European Medicines Agency (EMA), European Surveillance of Veterinary Antimicrobial Consumption 2014), In 2012, the overall consumption of polymyxins was approximately 600 times higher in food-producing animals compared to humans within the 19 member states of the EU/EEA, which reported complete data for both the animal sector and human medicine and after controlling for biomass (ECDC – European Centre for Disease Prevention and Control; EFSA – European Food Safety Authority and EMA – European Medicines Agency 2015). The latest data comparing the consumption of polymyxins among 28 EU/EEA member states show that the population-weighted mean of consumption is 340 times higher in food-producing animals compared to human medicine (ECDC – European Centre for Disease Prevention and Control, EFSA – European Food Safety Authority and EMA – European Medicines Agency 2017). However, polymyxin usage among countries varied dramatically within the sector. In addition, there was no association of the consumption of polymyxin between food-producing animals and humans within a country. As example, in 2014, Italy, Spain and Portugal reported the highest polymyxin usage in food-producing animals, whereas Greece, Ireland and United Kingdom consumed most of the polymyxin in human medicine after correction for biomass. Following the implementation of mandatory monitoring of AMR in zoonotic and commensal bacteria, a statistically significant positive correlation between polymyxin usage in animals and the emergence of polymyxin resistance in *E. coli* could be shown in 2014–2015 (ECDC – European Centre for Disease Prevention and Control, EFSA – European Food Safety Authority and EMA – European Medicines Agency 2017). In order to secure colistin (polymyxin E) as last-resort antibiotic for the human medicine and in accordance with the One Health approach, effort has been made to limit the use of polymyxins in food-producing animals. Overall, the sales of polymyxin in the veterinary sector were reduced by 54% in the EU between 2011 and 2018 (Fig. 1). The European Medicine Agency (EMA) recommended in their advice on colistin use in 2016 that EU member states with high polymyxin use should reduce the consumption in livestock below 5 mg/PCU by 2020 (European Medicines Agency (EMA) 2016). The latest data on polymyxin consumption by the EMA demonstrate that still six countries exceed this threshold in 2018, including Cyprus, Germany, Hungary, Poland, Portugal and Romania (European Medicines Agency – EMA, European Surveillance of Veterinary Antimicrobial Consumption 2020). However, the use of polymyxin cannot be completely abolished at present. Polymyxin is of great importance for the treatment of intestinal infections in pigs, poultry and veal calves caused by *Salmonella* spp. or *E. coli* due to its narrow bactericidal spectrum against Gram-negative bacteria (European Medicines Agency – EMA 2016).

### Human medicine

Colistin is used as last-line antimicrobial for treating infections with CRE that belong to MDR isolates and their occurrence has already been reported worldwide (Grundmann *et al*. 2010). Especially immunocompromised patients, e.g. in intensive care units or oncology wards, are susceptible to CRE infections (Satlin, Jenkins and Walsh 2014; Satlin and Walsh, 2017). Such infections are difficult to treat and with limited therapeutic options thereby leading to high morbidity and mortality rates (Parisi *et al*. 2015). The control of CRE infections can be achieved by two commercially available forms of colistin, colistin sulphate for oral and topical use and the prodrug CMS (syn. colistin methanesulphate, colistin sulphonyl methate and penta-sodium colistin methanesulphate) for parenteral use. Both drug forms can also be delivered by inhalation. CMS is microbiologically inactive and is less toxic than colistin sulphate. Following administration, CMS is hydrolyzed to colistin mediating the antibacterial effects. Besides colistin, also polymyxin B is licensed within the EU/EEA, but only for topical use. Colistin has been used for the treatment of infections at different body sites, e.g. bacteremia and ventilator-associated pneumonia. Especially in combination with other antibiotics such as tigecycline or carbapenems, colistin has been the preferred treatment option for carbapenemase-producing *Enterobacterales* (Li, Nation and Kaye 2019).

As reported for other antibiotics, colistin resistances emerged rapidly following its re-introduction in human medicine (Meletis *et al*. 2011; Capone *et al*. 2013). Several studies reported increasing colistin resistance rates in carbapenemase-producing *K. pneumoniae* in individual hospitals in Greece of 0% in 2007, 8.13% in 2008, 24.3% in 2009, 21.7% in 2013 and worryingly, an average of 40.4% between the years 2014 and 2016 in 15 participating hospitals (Meletis *et al*. 2011, 2015; Galani *et al*. 2018). In 2013, colistin resistance rates for CRE isolates for Spain and Italy were 31% and 43%, respectively (Monaco *et al*. 2014, Pena *et al*. 2014). A recent study reported current and slightly decreased colistin resistance rates in an Italian hospital of 20.1% in 2017, 31.2% in 2018 and 26.9% in 2019 (Basso *et al*. 2020). During the years 2007–2014, Norway identified a prevalence of chromosomally mediated colistin resistance of 21% among the tested human clinical isolates (Samuelsen *et al*. 2017). Germany reported a prevalence of 13.3% of colistin resistance among carbapenemase-producing *K. pneumoniae* isolated between 2011 and 2016 (Koppe *et al*. 2018). Especially outbreaks with colistin-/CREs are of great concern due to dramatically limited treatment options (Antoniadou *et al*. 2007; Mezzatesta *et al*. 2011; Mammina *et al*. 2012; Weterings *et al*. 2015; Haller *et al*. 2019). Alarmingly, the overall use of colistin in human medicine increased steadily in the EU between 2005 and 2018, probably due to an increase in MDR-resistant isolates (Fig. 1). However, there is a strong geographical heterogeneity regarding the colistin consumption among the European countries, with increased use in Greece, Malta and United Kingdom and no consumption in Austria, Portugal, or Germany (ESAC-Net interactive database, accessed 22nd October 2020). The choice of antibiotics to treat colistin-resistant XDR isolates depends on the infection type and body site, the susceptibility of the isolate as well as the pharmacokinetic/pharmacodynamic properties and potential side effects of the antimicrobial (Petrosillo, Taglietti and Granata 2019).

Noteworthy, the United Kingdom, Sweden and Greece have implemented a mandatory notification system of bloodstream infections caused by colistin-resistant bacteria (Anderson, Cecchini and Mossialos 2020).

In 2016, the WHO classified polymyxins into the group of critically important antimicrobials (CIA) with highest priority (HPCIA) for human medicine (World Health Organization (WHO) 2019). Complementary to this, the World Organization for Animal Health (OIE) included polymyxins in their list of veterinary antimicrobial agents into the class of high importance (World Organisation for Animal Health – OIE 2018). In 2017, in response to the steadily increasing AMR, the EU has stressed the One Health approach to combat antibiotic resistance in the animal and human medicine and to prevent transmission of zoonotic diseases (European Commission 2017). In 2019, The EMA updated its 2014 advice on the categorization of antibiotics used in veterinary medicine, which could pose a risk for human public health. Polymyxins are classified into category B (“Restrict”), which includes antimicrobials from the WHO HPCIA list, and should only be used in food-producing and companion animals for the treatment of infections when there is no alternative antibiotic from category C or D (EMA/688114/2020; European Medicines Agency – EMA 2019).

Since the monitoring of colistin resistance in *Enterobacterales* of livestock is mandatory, the surveillance of colistin-resistant isolates from human cases is still in its infancy. EU-wide data on colistin resistance in human isolates are collected only for the zoonotic agent *Salmonella*, but only a quarter of the EU/EEA member states reported to the ECDC (EFSA – European Food Safety Authority and ECDC – European Centre for Disease Prevention and Control 2019). The burden of AMR in Europe is assessed through the ECDC and the European Antimicrobial Resistance Surveillance Network (EARS-Net) in collaboration with national institutions. However, EARS-Net collects data from a limited number of bacterial species isolated from human blood and cerebrospinal fluid. As a result, infections affecting the urinary and respiratory tract caused by resistant bacteria, such as *E. coli* or *Klebsiella* spp., are not recorded. Moreover, colistin resistance in *Enterobacterales* is not assessed by EARS-Net as it is not included in the initial routine antimicrobial susceptibility testing and may only be examined by national laboratories (European Centre for Disease Prevention and Control – ECDC 2020). In 2019, the ECDC initiated a carbapenem-and/or colistin-resistant *Enterobacterales* (CCRE) project, which is a European network aiming to complement the phenotypic data collected by EARS-Net with WGS-based data, to address the needs described above. It is desirable however, to expand this effort to the surveillance data collected from the veterinary sector. As Tacconelli *et al*. pointed out, national and European-wide surveillance systems of AMR in livestock, the food chain and humans are very heterogeneous and need enhancement as well as improved multisectorial collaboration (Tacconelli *et al*. 2018). However, the most important basis for comparable data is the methodology for reliable colistin susceptibility testing. Standard broth microdilution (BMD) is the only recommended method by the European Committee on Antimicrobial Susceptibility Testing and Clinical and Laboratory Standards Institute for determination of the MIC values of colistin (European Committee on Antimicrobial Susceptibility Testing – EUCAST 2014), (Performance Standards for Antimicrobial Susceptibility Testing – CLSI 2020). However, this method is not automated, therefore time-consuming and difficult to incorporate into the routine of diagnostic laboratories. Other applied methodologies are broth macrodilution, disc diffusion, agar dilution, Etest, Rapid Polymyxin NP test and automated systems. For a reliable MIC determination, most likely two different systems should be combined such as an automated system and BMD, since so far all methods show specific weaknesses (Jayol *et al*. 2018; Garcia-Menino *et al*. 2020).

### GLOBAL USE OF COLISTIN: THE INCIDENCE AND MONITORING OF RESISTANCE

The global use of antibiotics in livestock animals is growing rapidly due to the growing global population and the increased production and consumption of animal protein as a result of increased incomes in fast-developing middle-income countries. The current OIE report states that still nine countries use colistin as growth promoter (World Organisation for Animal Health – OIE 2021). Contrary to the OIE report, Olaitan *et al*. (2021) pointed out that the majority of low- and middle-income countries still use colistin as feed additive. Discrepancies could be due to incomplete or inaccurate reporting to the OIE. In human medicine, the worldwide antibiotic consumption increased by 36% between 2000 and 2010, with Brazil, Russia, India, China and South Africa (BRICS] accounting for three-quarters of this increase, however, representing only 40% of the world's population. Thereof, the increase in colistin use in hospitals in BRICS countries for the same period corresponds to 13% (Laxminarayan *et al*. 2016).

**Figure 2.**
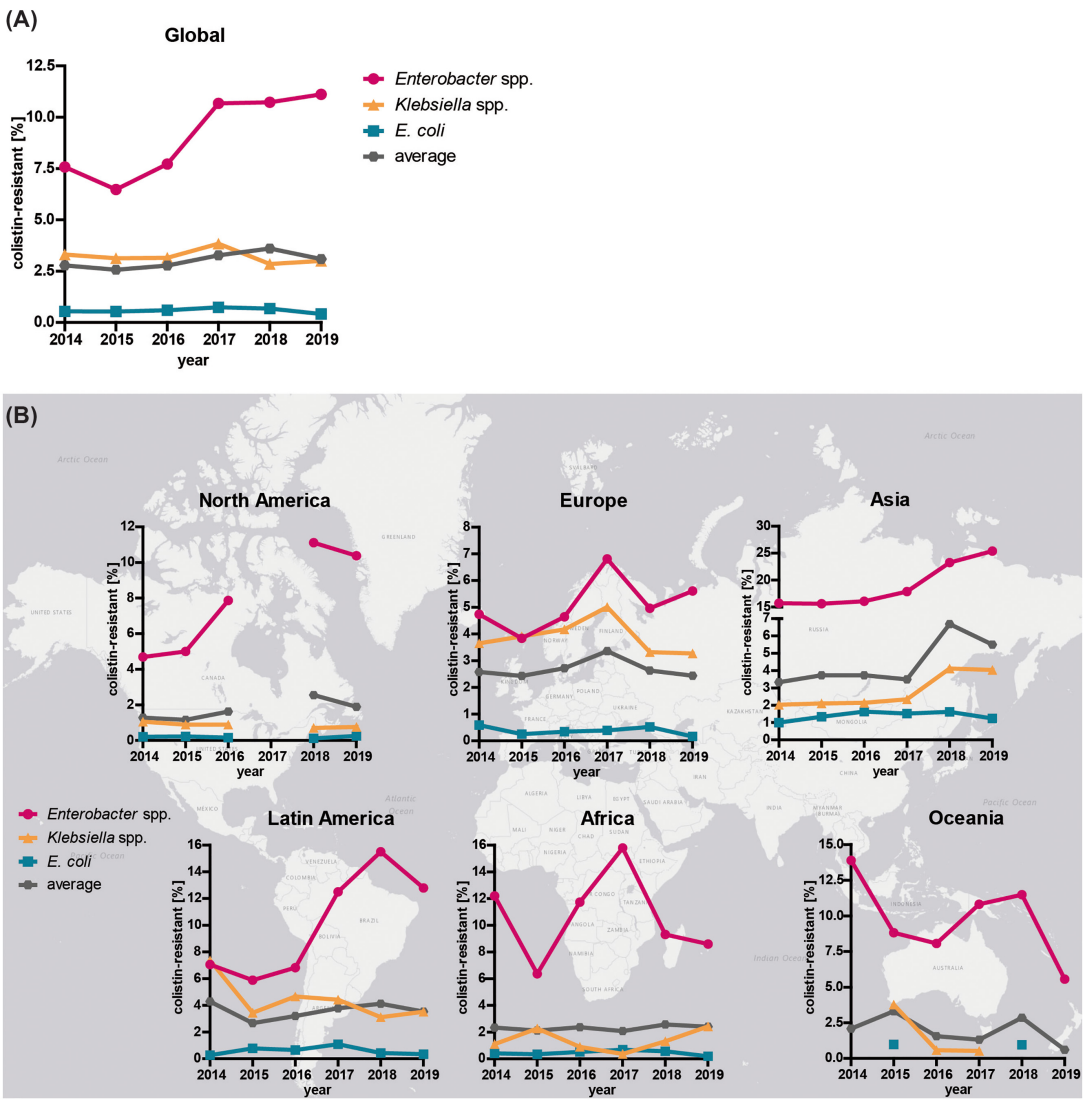
Global trends in colistin resistance in human clinical *Enterobacterales*. Data were obtained from the ATLAS database (https://atlas-surveillance.com, accessed February 2021), which includes data from the TEST (Tigecycline Evaluation Surveillance Trial) surveillance program, the AWARE (Assessing Worldwide Antimicrobial Resistance Evaluation) as well as INFORM (International Network for Optimal Resistance Monitoring) program. Resistance is shown as percentage from *Enterobacter* spp., *Klebsiella* spp. and *E. coli* using the data from all surveillance programs (ATLAS data source) and MIC values >2 mg/L according to the EUCAST breakpoint. **(A)** Global trend of colistin resistance among clinical *Enterobacter* spp., *Klebsiella* spp. and *E. coli* as well as combined genera from 2014 to 2019. **B**: Trends of colistin resistance in clinical *Enterobacterales* in different continents from 2014 to 2019. (A and B) Data reporting countries were: Europe: Austria, Belgium, Croatia, Czech Republic, Denmark, Finland, France, Germany, Greece, Hungary, Ireland, Italy, Latvia, Lithuania, Netherlands, Poland, Portugal, Romania, Russia, Spain, Sweden, Switzerland, Turkey, Ukraine and United Kingdom; North America: Canada, United States; Latin America: Argentina, Brazil, Chile, Colombia, Costa Rica, Dominican Republic, Guatemala, Mexico, Panama and Venezuela; Asia: China (incl. Hong Kong and Taiwan), Japan, Korea, South, Malaysia, Philippines and Thailand; Africa: Israel, Jordan, Kenya, Kuwait, Morocco, Nigeria, Qatar, Saudi Arabia and South Africa; Oceania: Australia and New Zealand.

The prevalence of the global colistin resistance among human clinical *Enterobacterales* isolated between 2012 and 2013 was 1.6% with a regional distribution of the resistance in Europe (1.8%), North America (1.3%), Latin America (1.5%), Middle East-Africa (1.4%) and the Asia–Pacific (1.3%) (Bradford *et al*. 2016). The most abundant resistant genera was *Enterobacter* spp., followed by *K. pneumoniae* and *E. coli*. By using the surveillance data from the ATLAS database, the global incidence of colistin-resistant human clinical *Enterobacterales* increased between 2014 and 2019 from 2.6 to 3.6%, showing a regional distribution of 2.4–3.4% in Europe, 1.2–2.6% in North America, 2.7–4.3% in Latin America, 3.3–6.7% in Asia, 2.1–2.6% in Africa and 0.6–2.7% in Oceania (Fig. 2 ).

**Figure 3.**
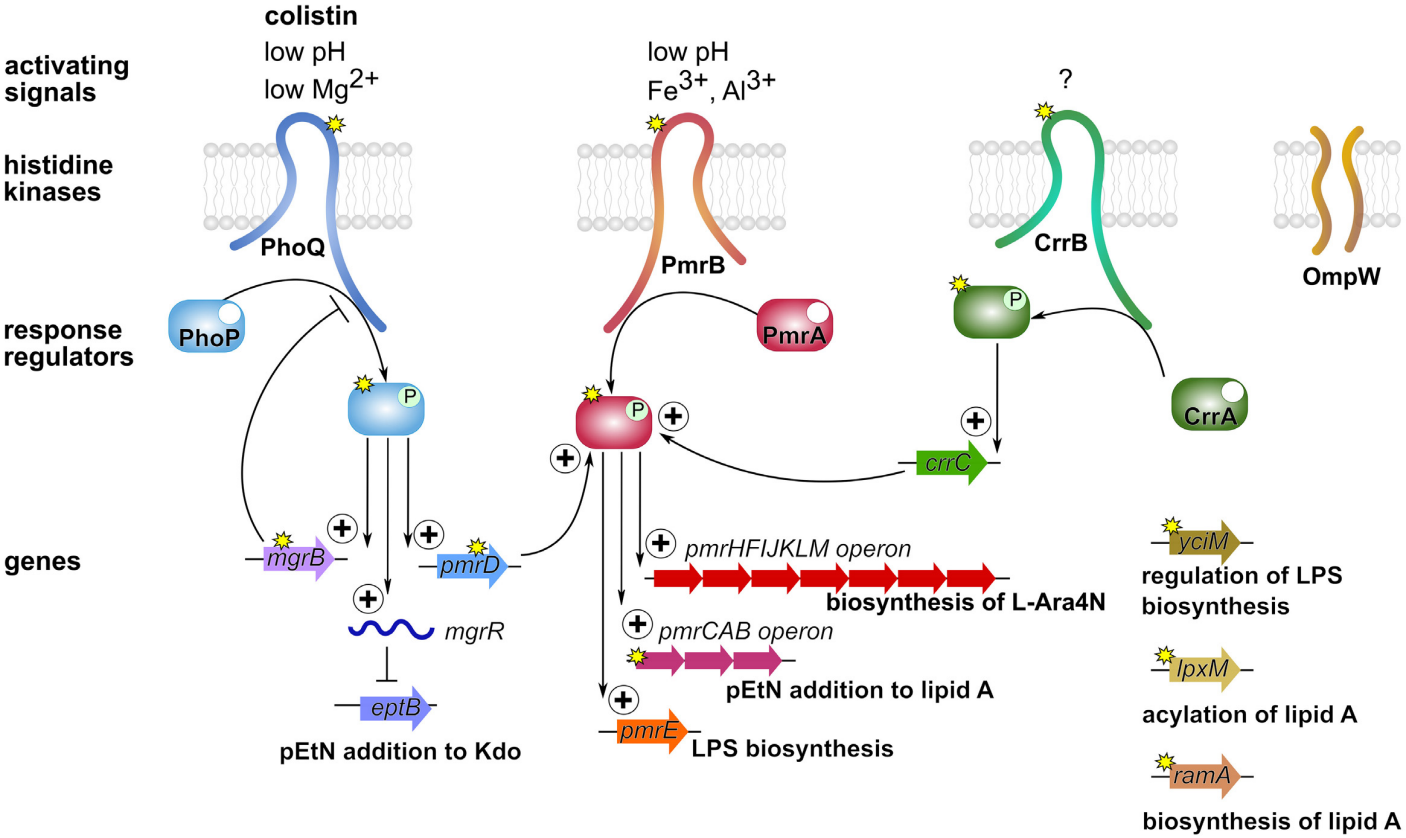
Regulatory network of LPS-modifying proteins involved in colistin resistance in *Enterobacterales*. The PhoPQ TCS is activated by low Mg^2+^ concentrations, low pH and the presence of antimicrobials peptides, such as colistin, leading to the expression of the regulator MgrB, the adaptor protein PmrD and the sRNA *mgrR*. MgrB exerts negative feedback on PhoQ, while mutations in MgrB typically result in the constitutive activation of the PhoPQ TCS. The sRNA *mgrR* impedes the expression of EptB. The adaptor protein PmrD activates the PmrAB TCS leading to the expression of multiple target genes responsible for LPS biosynthesis and modification. PmrA also becomes activated by the CrrAB TCS via the adaptor protein CrrC. Gain-of function mutations in CrrB can also result in the activation of gene expression of the *pmrHFIJKLM* operon without involvement of the PmrAB TCS. In addition, mutations in the proteins YciM and LpxM have been found to confer colistin resistance. The plus symbol indicates positive regulation and the yellow star highlights alterations in proteins/genes, which may lead to colistin resistance.

#### Asia

##### China

China is a leading consumer and producer of colistin worldwide. Between 2011 and 2015, about 2875 metric tons of colistin were used in food-producing animals annually (Shen *et al*. 2016). In November 2016, the Ministry of Agriculture announced the withdrawal of colistin as feed additive to promote animal growth, which took into effect in April 2017 (Walsh and Wu, 2016). Thereafter, the production of colistin sulfate premix decreased steadily, from 27 170 tons in 2015 to 2497 tons in 2018 with the most significant decrease between 2016 and 2017 (Wang *et al*. 2020). Polymyxin B and colistin became available for the use in humans in October 2017 and December 2018, respectively (Table S2, Supporting Information).

##### India

Data from 2016 on colistin shipments showed strong exports of the antimicrobial from China to India, South Korea and Vietnam. Antimicrobial use in chickens is estimated to increase 5-fold by 2030 compared with 2013 (Davies and Walsh, 2018). However, data on colistin-resistant *Enterobacterales* from livestock animals are lacking. The Indian Antimicrobial Resistance Surveillance & Research Network collects data from AMR profiles from clinically relevant pathogens. Between 2016 and 2018, the prevalence of colistin resistance in *E. coli* and *K. pneumoniae* causing hospital-acquired infections was 1.1% and 8%, respectively (Walia *et al*. 2019). Alarmingly, 70 and 83.3% of *E. coli* and *K. pneumoniae* isolated from hospital wastewater were resistant to colistin (Bardhan, Chakraborty and Bhattacharjee 2020). Another study analyzing the presence of colistin-resistant bacteria on raw food, including meat, fish and vegetables, found that 46.4% of the samples contained colistin-resistant bacteria, of which the most prevalent species were *E. coli* and *Klebsiella* species (Ghafur *et al*. 2019). Remarkably, since July 2019 the Indian Union Ministry of Health and Welfare has banned colistin for the use in food-producing animals, which goes beyond the use as growth promoter and includes the prohibition for pro-and metaphylaxis purposes (section 26A of Drugs and Cosmetics Act, 1940; Table S2, Supporting Information).

In 2019, the rate of colistin resistance in *Enterobacterales* isolated from clinical specimen in Asia was 25.4% for *Enterobacter* spp., 4.0% for *Klebsiella* spp. and 1.2% for *E. coli* (Fig. 2). Only few studies from Asia investigated chromosome-mediated colistin resistance in *Enterobacterales*, which mainly comprises the analysis of clinical *Klebsiella* spp. isolates from Lebanon, Taiwan, Turkey, Iran and United Arab Emirates (Cheng *et al*. 2015; Okdah *et al*. 2017; Can *et al*. 2018; Moubareck *et al*. 2018; Dagher *et al*. 2019; Jafari *et al*. 2019). Described resistance mechanisms were disruption of the *mgrB* gene, as well as missense mutations in MgrB, PhoQ and PmrB. A total of two studies from South Korea and Japan found missense mutations in PhoPQ and PmrAB two-component system in clinical *E. cloacae* strains, but their contribution to colistin resistance was not confirmed (Hong and Ko, 2019; Uechi *et al*. 2019).

A systematic review covering southeast Asia identified reports on chromosomal mutations in *mgrB* in *Klebsiella* spp. isolates (Malchione *et al*. 2019). Susceptibility to colistin is not routinely tested in these countries, resulting in limited data and underestimation of resistance levels. Colistin resistance has also been described for *Salmonella* spp. strains from chicken and turkey in South Korea and Taiwan, but the mechanism of resistance has not been specified (Yeh *et al*. 2018; Seo *et al*. 2019).

#### Australia

Australia has approved polymyxin B but not colistin or its formulations for the use in livestock animals (Commonwealth of Australia 2018; Table S2, Supporting Information). Studies analyzing resistant bacteria from poultry and egg layer flocks found neither colistin-resistant *Enterobacterales* nor mobilizable genes conferring colistin resistance (Abraham *et al*. 2019; Bean *et al*. 2020; Veltman *et al*. 2021). Colistin and polymyxin B are authorized for the use in human medicine (Australian Commission on Safety and Quality in Health Care 2019). The Australian National Alert System for Critical Antimicrobial Resistances (CARAlert) monitors only transmissible colistin resistance in clinical *Enterobacterales* of human origin. Only a few studies reported infections caused by colistin-resistant *Enterobacterales* in humans. In accordance with the data provided by the ATLAS database, the percentage of resistant isolates collected between 2007 and 2016 was 2.1% (Fig. 2; Ellem *et al*. 2017). 98% of the isolates analyzed were negative for *mcr-1*, suggesting that they carry either a different *mcr*-gene or a chromosomal mutation (Ellem *et al*. 2017).

#### North America

##### USA

Colistin has been approved but never been marketed for the use in food-producing animals in the United States (U.S.; https://www.center4research.org/8094-2/, accessed June 2021). Since 2009, polymyxin B has been approved and used as antimicrobial (Table S2, Supporting Information; U.S. Food and Drug Asministration – FDA 2016; https://animaldrugsatfda.fda.gov/adafda/views/#/home/searchResult, accessed June 2021). Therefore, the presence of colistin resistance in *Enterobacterales* remains very low with a prevalence of 0.1% in animals at slaughter and 0.02% from animal meat (Meinersmann *et al*. 2017; Wang *et al*. 2020). Within the National Antimicrobial Resistance Monitoring System (NARMS), which collects bacteria from humans, animals and food, isolates are not tested for susceptibility to colistin. The latest report states that NARMS sequenced the genomes of 55 000 *Salmonella* and *E. coli* isolates and found only *mcr-9.1* in 55 *Salmonella* strains from humans, animals and meat, as well as in five *E. coli* isolated from meat (National Antimicrobial Resistance Monitoring System – NARMS 2018, Wang *et al*. 2020). Macesic *et al*. (2020) found chromosomal alterations in colistin resistance genes of *K. pneumoniae* in 36% of patients. According to the United States Committee on Antimicrobial Susceptibility Testing, reliable detection of colistin resistance in human clinical isolates is lacking due to the use of test systems (e.g. Etest, disk diffusion) other than the recommended reference method, and the absence of colistin in most automated test systems, which accounts for over 90% of the U.S. test results (Pogue *et al*. 2020).

##### Canada

The Public Health Agency of Canada has announced that polymyxin B is approved for the use in livestock animals (Canadian Ministry of Agriculture 2019). An EMA report from 2016 states that colistin was not approved for the use in veterinary medicine. Unfortunately, we were not able to conclusively clarify the statement with the source of the Public Health Agency of Canada indicated in the EMA report or with other sources (European Medicines Agency – EMA 2016). However, a loophole in the regulation gave farmers the opportunity to import and use unlicensed, non-prescription antimicrobial combinations of third-generation cephalosporins and penicillin containing colistin in their livestock, which were used very frequently on dairy farms (Saini *et al*. 2012; Webb *et al*. 2017). In 2016, the loophole of “importation for own use” has been recognized and regulatory changes have been proposed that would prohibit those practices (https://canadagazette.gc.ca/rp-pr/p1/2016/2016-07-02/html/reg2-eng.html, accessed August 2021). In addition, polymyxin B and colistin are used in the human medicine (Public Health Agency of Canada 2018). The Canadian Ward Surveillance Study monitors AMR in multiple pathogens in 15 hospitals across the country. Across samples collected from 2007 to 2016 the prevalence of colistin resistance in *E. coli*, *Klebsiella* spp. and *Enterobacter* spp. was 0.2%, 5.8% and 18.1%, respectively (Zhanel *et al*. 2019). Unfortunately, the trend of colistin resistance development within the period was not calculated for the corresponding isolates (Lagace-Wiens *et al*. 2019).

In 2019, the overall prevalence of colistin resistance among clinical *Enterobacterales* in North America was 10.4% for *Enterobacter* spp., 0.8% for *Klebsiella* spp. and 0.3% for *E. coli* (Fig. 2).

#### Central and South America

The Pan American Center PANAFTOSA oversees AMR in zoonotic agents, while AMR in community-and hospital-acquired pathogens is monitored by the ReLAVRA (Latin American Network for Antimicrobial Resistance Surveillance), which covers 19 member states. However, uniform data on the overall colistin susceptibility in *Enterobacterales* of animal and human origin from member states are not available. Colistin is no longer approved for the use in livestock in Nicaragua, Costa Rica, Peru, Paraguay and Argentina (Table S2, Supporting Information). Besides Guatemala, El Salvador, Panama, Venezuela, Guyana, Suriname and French Guiana, which did not provide information, colistin is approved as a therapeutic in the other countries of Latin America (unpublished, personal communication with PANAFTOSA, February 2021). A study analyzing colistin resistance in *Enterobacterales* collected from hospitals in six South American countries between 2015 and 2017 found a prevalence of resistance of 5.6% in *E. cloacae*, 4.9% in *K. pneumoniae*, 1.4% in *K. aerogenes*, 0.9% in *K. oxytoca* and 0.8% in *E. coli*, which is similar to the data provided by the ATLAS database (Stone and Ponce-de-Leon, 2020; Fig. 2). In 2019, resistance to colistin among human clinical *Enterobacter* spp., *Klebsiella* spp. and *E. coli* was 12.8%, 3.5% and 0.3% respectively (Fig. 2).

##### Brazil

Besides China, Brazil is one of the largest poultry producers and exporter globally (Food and Agriculture Organization of the United Nations – FAO 2020). In 2016, Brazil prohibited the use of colistin as feed-additive in food-producing animals (Brazil. Governmental Normative Instruction IN-45 2016). However, due to the lack of sufficient studies it is not possible to assess the colistin resistance levels in livestock animals. Morales *et al*. (2012) reported a prevalence of 6.3% of colistin-resistant *E. coli* in swine samples. The colistin resistance rate among human clinical *Enterobacterales* increased from 6.6% in 2010 to 9.9% in 2014 using samples from nine hospitals of São Paulo, Brazil (Rossi *et al*. 2017). Another hospital reported a resistance rate of 5% in *Enterobacterales* mediated by chromosomal mutations (2%) and *mcr*-genes (3%; Rocha *et al*. 2020).

##### Colombia

A study from Colombia analyzing colistin-resistant clinical isolates found that only 2.3% of the *Enterobacterales* carried *mcr-1*, suggesting that the majority of isolates have a chromosomal mutation that leads to colistin resistance (Saavedra *et al*. 2017). However, analyzed isolates contain the serovars *S*. Enteritidis and *S*. Dublin, which seem to exhibit increased intrinsic resistance to colistin, eventually leading to an overestimation of the overall number of resistant strains.

The majority of studies analyzing the prevalence of colistin resistance in *Enterobacterales* in Latin American countries focus on the presence of *mcr-*genes rather than chromosomal mutations. In total, two studies described disruptions of the *mgrB*-gene leading to colistin resistance in clinical *K. pneumoniae strains* isolated in Argentina and Uruguay (Alvarez *et al*. 2018; Escalante *et al*. 2020). Reports from other Latin American countries, such as Mexico, Argentina, Chile, Peru, Ecuador, Venezuela and Uruguay focus exclusively on the identification of *mcr-1* in *E. coli* obtained from livestock, companion animals and human clinical specimen (Delgado-Blas *et al*. 2016; Ortega-Paredes, Barba and Zurita 2016; Dominguez *et al*. 2017, 2019 Garza-Ramos *et al*. 2018; Gutierrez *et al*. 2019; Merida-Vieyra *et al*. 2019; Rumi *et al*. 2019; Coppola *et al*. 2020; Loayza-Villa *et al*. 2020; Papa-Ezdra *et al*. 2020).

#### Africa

Livelihoods of 250–300 million people in Africa is financed by animal husbandry, and the use of colistin in livestock remained largely unregulated (Van *et al*. 2020). For example, in 2015 in Morocco, colistin was the most frequently (27.85% of treatments) used antimicrobial for treatments in the broiler sector and the second most commonly used antimicrobial by active ingredient. Furthermore, colistin was overdosed in most of the administrations (Rahmatallah *et al*. 2018). Similarly, colistin resistance in *E. coli* from South African poultry increased steadily from 3.9% in 2009 to 12.08% in 2015 (Theobald *et al*. 2019). Following the prohibition of colistin as a feed additive in 2016, resistance levels in avian *E. coli* in South Africa decreased to 1.77% (Table S2, Supporting Information). End of 2017, the Africa CDC founded the Anti-Microbial Resistance Surveillance Network (AMRSNET) for the monitoring of resistant organism in the animal and human health sector. Due to the recent establishment of the network, data regarding the overall prevalence of colistin-resistant *Enterobacterales* in Africa are not yet available. Figure 2 shows resistance levels of 8.6% for *Enterobacter* spp., 2.4% for *Klebsiella* spp. and 0.19% of *E. coli* clinical isolates in 2019. A systematic review by Olowo-okere and Yacouba identified studies regarding colistin-resistant bacteria from Algeria, Egypt, Tunisia, South Africa, Libya, São Tomé and Príncipe and Nigeria. Overall, colistin resistance was most frequently described for *E. coli* isolates obtained from human clinical samples. Furthermore, studies reported both chromosomal and plasmid-mediated resistance mechanisms, of which plasmid-mediated colistin resistance was the most prevalent, accounting for up to 72.2%. Mutational changes were found in *pmrA/B* of *E. coli* and *K. pneumoniae* and additionally in *mgrB* of *K. pneumoniae* (Olowo-okere and Yacouba, 2020).

A study performed in South Africa analyzing colistin resistance mechanisms in human clinical strains collected between 2016 and 2017 notified a prevalence of *mcr-1* in 55% of *E. coli* and 71% of *Klebsiella* isolates. The analysis of the chromosomal-mediated colistin resistance showed that genetic alterations occurred predominantly in *pmrB* and *mgrB* of *E. coli* and *Klebsiella* isolates, respectively (Snyman *et al*. 2021). However, colistin-resistant *Enterobacterales* were isolated not only from hospitalized patients but, more worryingly, also from healthy hotel employees in Zanzibar, Tanzania, with a prevalence of 59.3%. Overall, 55% of the colistin-resistant *E. coli* isolates carried *mcr*-1, whereas none of *K. pneumoniae* harbored *mcr*-1 to *mcr*-8 (Budel *et al*. 2019). In addition to animals and humans, two reports from South Africa and Tunisia demonstrated colistin resistance among cefotaxime-resistant *E. coli* (76.5%) as well as ESBL-producing *Enterobacterales* (10.8%) that were *mcr*-negative, isolated from waste water (Adegoke *et al*. 2020; Hassen *et al*. 2020)

#### Transmission of colistin-resistant *Enterobacterales* in a One Health perspective

**Figure 4.**
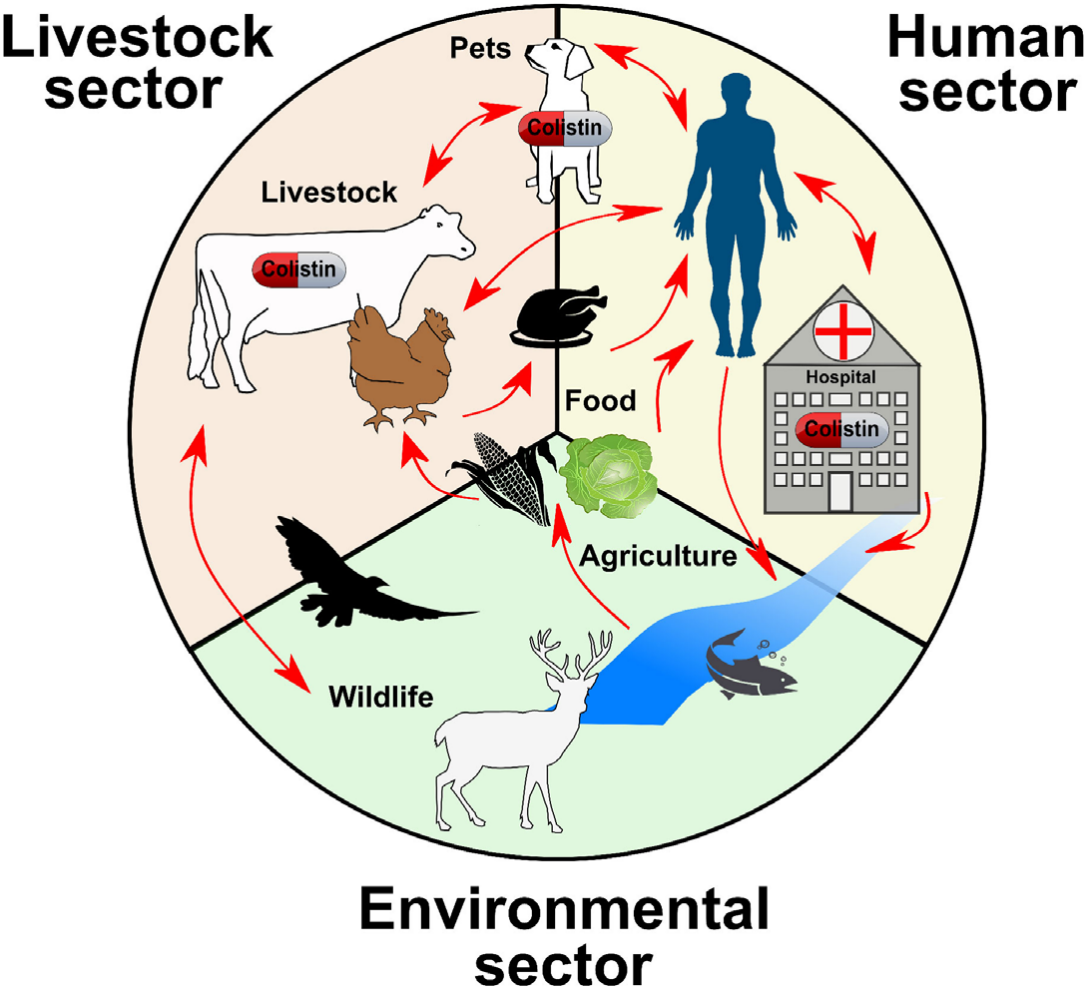
Possible transmission routes of colistin-resistant *Enterobacterales*. Colistin-resistant *Enterobacterales* emerge as a result of the use of colistin in the livestock sector, in animal clinics and the hospitals. Resistant isolates can disseminate between different areas of life, which is indicated by the red arrows.

Globalization connects the different areas of life but also facilitates the spread of AMR. Humans and animals, including domestic animals and wildlife, continuously interact with each other and share often the same habitat. The excessive use of colistin in animals resulted in the selection for resistance affecting both human and animal health. Overall, three different pathways for the transmission of resistant bacteria can be recognized: (i) transmission between animals and humans, (ii) transmission to humans/animals via contaminated food and (iii) transmission via the environment (Fig. 4). The transmission of colistin-resistant bacteria occurs in direct contact among animals, humans and between them (Budel *et al*. 2020). Especially human individuals in constant contact with animals, e.g. farm workers and veterinarians, are at greater risk of acquiring resistant microorganisms (Marshall and Levy, 2011). Slaughterhouses and farms are main places of inter-species transmission, where colistin-resistant bacteria are transferred from animals to humans in the event of contamination during the slaughter process, but also may enter the food chain and the environment via sewage. Chromosome-mediated colistin-resistant *E. coli* were found in livestock in Europe, Asia and Africa (Rebelo *et al*. 2018; Kim *et al*. 2019; Budel *et al*. 2020; Massella *et al*. 2020).

Backyard livestock and small family farms are common husbandry systems for food-producing animals in Asia where antimicrobials are often overused resulting in high resistance levels (Hallenberg *et al*. 2019; Kawahara *et al*. 2019). Furthermore, poor hygiene and close contact with farm staff facilitates the transmission of colistin-resistant *Enterobacterales* from animals to humans (Trung *et al*. 2017). Noteworthy, transmission of colistin-resistant *E. coli* also occurs between animals on integrated poultry-fish farming systems where chickens are kept over fish ponds and feces is excreted into the ponds. In 2017, the most common antimicrobial used in poultry-fish farms in Myanmar was Octamix (amoxicillin and colistin sulfate) in over 40% of poultry flocks and 6% of *E. coli* isolated from feces were resistant to colistin (Gibson *et al*. 2020).

Besides farm animals, also companion animals are increasingly discussed to serve as vehicle for a potential bacterial transmission (Joosten *et al*. 2020; Marin *et al*. 2021). Notably, a study from Switzerland uncovered the contamination of surfaces in companion animal clinics with colistin-resistant *Enterobacterales* and worryingly, the colonization of employees with resistant strains (Schmidt *et al*. 2020). Although farm workers, veterinarians and pet owners represent only a limited number of people having close contact with animals, they still provide an entry point for the transmission of resistant *Enterobacterales* into the community and hospitals. Several studies demonstrated precisely the spread of colistin-resistant pathogens, in hospital outbreaks. *Klebsiella* spp. in particular, with mutations in chromosomally encoded genes, is a major cause of the clonal dissemination in clinical settings around the world (Mezzatesta *et al*. 2011; Mammina *et al*. 2012; Goel *et al*. 2014; Giani *et al*. 2015; Weterings *et al*. 2015; Jayol *et al*. 2016; Kocsis *et al*. 2017; Avgoulea *et al*. 2018; Guducuoglu *et al*. 2018; Haller *et al*. 2019). But also clinical *Enterobacter* spp. isolates harboring colistin resistance have been described (Hong, Lee and Ko 2018).Transmission of resistant bacteria during surgery, the acquisition from hospital surfaces, or solely the transfer between patients and health care workers via hand contact may increase the risk for the dissemination of resistant pathogens in hospital settings (as already shown for methicillin-resistant *Staphylococcus aureus* or *A. baumannii*; Blanco, O'Hara and Harris 2019).

Resistant bacteria can also reach the consumer through the consumption of contaminated food, which displays a far more complex route of transmission. Sources for foodborne transmission are the consumption of animal-related products, such as meat and fish. Colistin-resistant *K. pneumoniae* lacking *mcr* genes were isolated from fish and poultry meat from Europe, Africa and Asia (Ghafur *et al*. 2019; Diaz-Jimenez *et al*. 2020; Chaalal *et al*. 2021). Furthermore, colistin-resistant *S*. Abony strains with mutations in chromosomal genes were located in fish farms (Antunes *et al*. 2018). A less noticed vehicle for transmission of colistin-resistant bacteria are vegetables and seafood, where the consumption in the raw state may display a greater risk (Ghafur *et al*. 2019). In addition, international food trade may facilitate the introduction and spread of colistin-resistant *Enterobacterales*. Finally, resistant bacteria can also disseminate via waste material, such as sewage, contaminating the environment (Savin *et al*. 2020). Especially water is an efficient route for bacterial transmission into nature and wild life. Colistin-resistant *Enterobacterales* have been found in several species of wild animals, such as mice, deer and sea lions across several countries (Wasyl *et al*. 2018; Hernandez-Castro *et al*. 2020; Skarzynska *et al*. 2020; Zanardi *et al*. 2020).

Altogether, these findings highlight the importance of national monitoring programs and global routine surveillance of colistin resistance in zoonotic bacteria of animal, food and human origin, which provides scientific data for the assessment of AMR burden as well as for strategic interventions.

#### Colistin-resistant *Enterobacterales* and associated sequence types

Studies regarding AMR in livestock animals focused primarily on *E. coli* and *Salmonella*. As early as 1975, it was determined that the administration of sub-therapeutic quantities of antibiotics is sufficient to develop resistance in *E. coli* in the gastrointestinal tract of chicken. Strikingly, the farm workers also acquired resistant *E. coli* in their intestine (Levy, Fitzgerald and Macone 1976). Due to the uninterrupted use, colistin resistance has been continuously reported in *E. coli* and *Salmonella* isolated from farm animals.

Especially in healthcare setting, CRE, predominantly *K. pneumoniae* and *Enterobacter* spp., emerged in recent years as a major threat in the group of antibiotic-resistant pathogens (Peleg and Hooper, 2010; Chavda *et al*. 2016). Mortality rates are high due to limited treatment options and successful dissemination of certain strains. CRE have been reported from several countries, but especially the Mediterranean countries, such as Greece, Italy, Malta and Israel, report the rapid spread of endemic clones in many hospitals (Leavitt *et al*. 2007; Samra *et al*. 2007; Capone *et al*. 2013; Glasner *et al*. 2013; Albiger *et al*. 2015). In Europe, 23 European countries report a worsened epidemiological situation of CRE between the years 2010 and 2018 (Brolund *et al*. 2019). Colistin, together with tigecycline and gentamicin, is among the few antimicrobial available to treat infections with CRE (Petrosillo *et al*. 2013). Therefore, the emergence of resistance to colistin and other last-option antimicrobials especially in, but not restricted to, CRE is important to monitor.

#### Escherichia coli

*Escherichia coli* is a part of the normal intestinal microbiome in animals and humans. However, *E. coli* is also the most prevalent bacterial agent causing community- and hospital-acquired infections such as urinary tract and bloodstream infections. To date, no association could be established between colistin resistance in clinical isolates and consumption in human medicine due to lack of data on *E. coli* (ECDC – European Centre for Disease Prevention and Control, EFSA – European Food Safety Authority and EMA – European Medicines Agency 2017). In contrast, in food-producing animals, a strong positive correlation was revealed between the resistance to and the consumption of colistin. The European Food Safety Authority (EFSA) and the European Centre for Disease Prevention and Control (ECDC) identified in their report from 2019a prevalence of colistin resistance of 0.3% and 0.8% in fattening pigs and calves, respectively (EFSA – European Food Safety Authority and ECDC – European Centre for Disease Prevention and Control 2019). Data for poultry were only available for 2015, when colistin resistance was reported in 1.7% of broilers and 5.7% of turkeys (EFSA – European Food Safety Authority and ECDC – European Centre for Disease Prevention and Control 2018). Only Italy reported on a voluntary basis data regarding colistin resistance in meat products, which was 5.3% in pig meat and 3.1% in bovine meat (EFSA – European Food Safety Authority and ECDC – European Centre for Disease Prevention and Control 2019).

**Figure 5.**
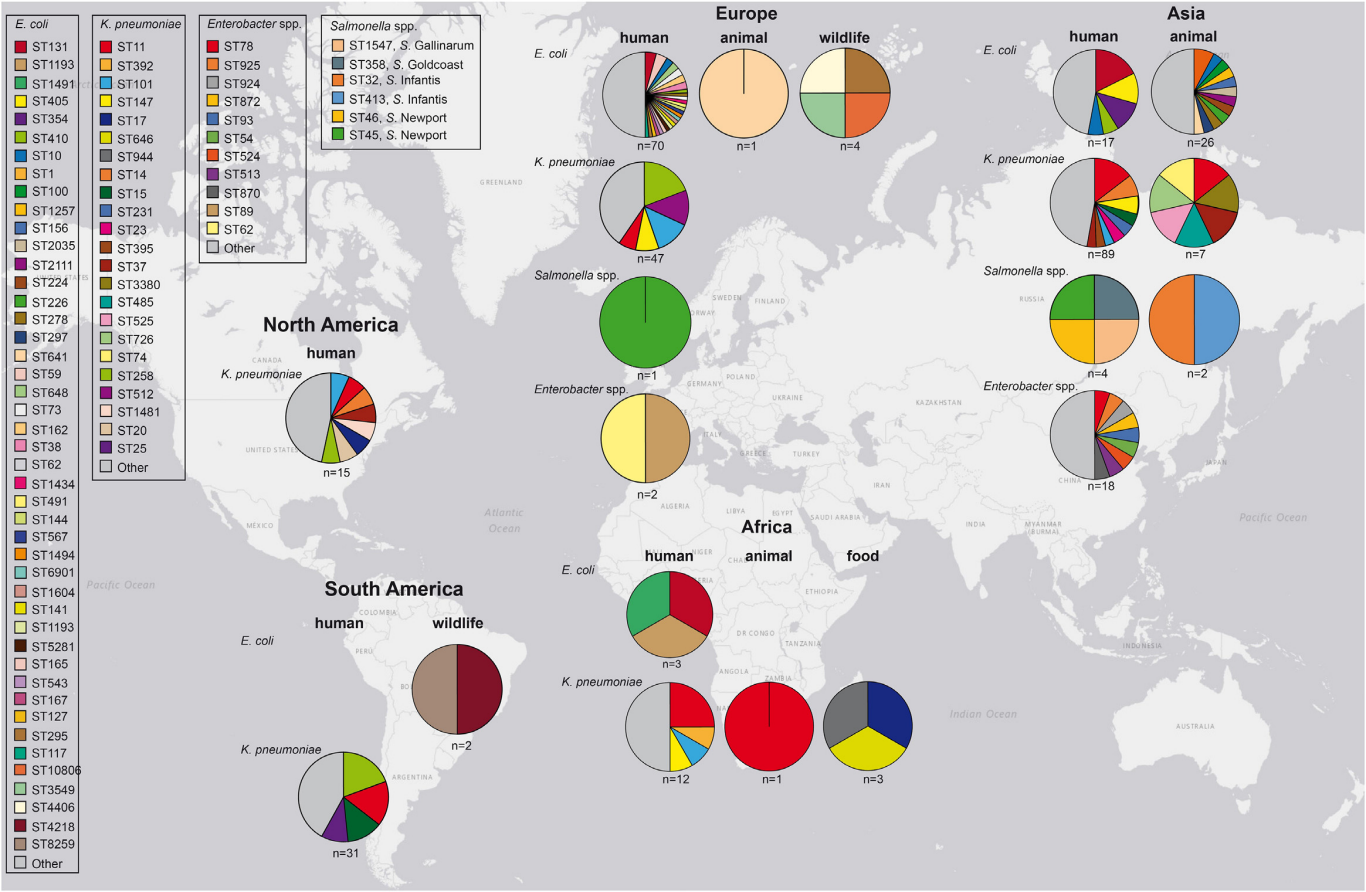
Landscape of *Enterobacterales* sequence types associated with chromosomal mutations leading to colistin resistance. Worldwide prevalence of chromosomal-mediated colistin resistance in different sequence types from *E. coli*, *K. pneumoniae*, *Enterobacter* spp. and *Salmonella* spp. isolated from human, animal, food and wildlife. Detailed information is given in Table S2 (Supporting Information) in addition with information regarding sequence types of each species associated with *mcr*-gene

Certain sequence types occur repeatedly among *E. coli* strains isolated from humans and food-related animals and worryingly, belong to high-risk clones, which display a major health concern. Several studies from Europe and Asia identified the *E. coli* ST10 as a common isolate in livestock animals and humans with bloodstream or urinary tract infections, which is globally distributed and able to develop chromosomally encoded colistin resistance (Fig. 5 and Table S3, Supporting Information; Luo *et al*. 2017; Kim *et al*. 2019; Janssen *et al*. 2020). *Escherichia coli* ST131 is currently the most important human clone worldwide and is frequently associated with chromosomal colistin resistance (Sato *et al*. 2016, 2018; Luo *et al*. 2017; Dafopoulou *et al*. 2020; Dagher *et al*. 2020; Snyman *et al*. 2021). Human clinical *E. coli* of the pandemic clonal group ST131 with chromosomal mutations in colistin-resistance determinants were reported predominantly from Asia and Europe, but also from Africa (Fig. 5 and Table S3, Supporting Information).

The review of the scientific literature revealed a predominant number of studies analyzing the presence of *mcr*-genes in *E. coli* strains obtained from livestock animals and animal products, as opposed to only a few reports dealing with chromosome-mediated colistin resistance in those isolates.

#### Klebsiella pneumoniae

*Klebsiella pneumoniae* colonizes the respiratory and gastrointestinal tract of humans (Bagley, 1985; Martin and Bachman, 2018). XDR and PDR *Klebsiella* spp. strains are major cause of healthcare-associated infections and outbreaks leading to difficult-to-treat diseases, e.g. lower respiratory tract infections, urinary tract infections and bloodstream infections (Martin and Bachman, 2018). An example of this are carbapenemase-producing *K. pneumoniae* strains belonging to pandemic clones, such as ST11, ST147, ST258 and ST525, which have been reported to acquire resistances to numerous unrelated antimicrobial agents. (Comandatore *et al*. 2013; Monaco *et al*. 2014; Pena *et al*. 2014; Giani *et al*. 2015; Oteo *et al*. 2016; Samuelsen *et al*. 2017; Diaz-Jimenez *et al*. 2020; Gentile *et al*. 2020). Colistin resistance caused by chromosomal alterations in isolates with the above mentioned sequence types is reported from countries in Africa, Asia, Europe as well as North and South America from human clinical specimen (Fig. 5 and Table S3, Supporting Information; Cannatelli *et al*. 2014; Jaidane *et al*. 2018; Teo *et al*. 2019; Macesic *et al*. 2020; de la Cadena *et al*. 2021). Additionally, colistin resistant ST11 is also isolated from animal sources from Africa and Asia (Pishnian, Haeili and Feizi 2019; Budel *et al*. 2020). The prevalence of chromosome-mediated colistin resistance among carbapenemase-producing isolates ranges from 6 to 80% among EU member states (Pena *et al*. 2014; Bonura *et al*. 2015; Jayol *et al*. 2016; Oteo *et al*. 2016; Otter *et al*. 2017; Samuelsen *et al*. 2017; Hamel *et al*. 2020). High colistin resistance rates among carbapenem-resistant klebsiellae, ranging from 27 to 61%, were also reported from Asia, South Africa and South America (Sampaio and Gales, 2016; Jafari *et al*. 2019; Al-Zalabani *et al*. 2020; Kopotsa, Mbelle and Sekyere 2020; Shankar *et al*. 2021). Notably, a strong correlation was found between emerging polymyxin resistance in *K. pneumoniae* isolates and consumption of polymyxin in the hospital sector (ECDC – European Centre for Disease Prevention and Control, EFSA – European Food Safety Authority and EMA – European Medicines Agency 2017).

In contrast to studies regarding colistin-resistant *E. coli* isolates from the livestock sector, most of the publications about human-pathogenic colistin-resistant *Klebsiella* strains analyzed the contribution of mutational changes in chromosomal genes.

#### Salmonella enterica

Non-typhoidal *Salmonella* is a major cause of food poisoning resulting in gastrointestinal infections that range from asymptomatic to clinically severe illness. In 2018, salmonellosis was the second most common gastrointestinal infection in the EU/EEA (European Food Safety Authority – EFSA) and European Centre for Disease Prevention and Control – ECDC 2019). The predominant risk factor for the acquisition of *Salmonella* is the consumption of contaminated food, such as meat, eggs, vegetables and dairy products. The largest proportion of colistin-resistant *Salmonella* strains derived from livestock animals in the EU were found in cattle with a prevalence of 14.5% (EFSA – European Food Safety Authority and ECDC – European Centre for Disease Prevention and Control 2019). In calf carcasses under 1 year of age, 3.7% of *Salmonella* isolates were resistant to colistin. However, all resistant strains derived from cattle and calf carcasses belong to serovar *S*. Dublin. The ECDC and EFSA joint report from 2019refers to a study from 2012, which suggests that the serovars *S*. Dublin and *S*. Enteritidis exhibit increased intrinsic resistance levels to colistin. However, only two chromosomal genes were analyzed in the mentioned study and this review summarizes additional genes involved in colistin resistance (Agerso *et al*. 2012). In addition, other yet unidentified mechanisms or mutations in these serovars could mediate colistin resistance.

Furthermore, EU member states reported colistin resistance in 1.9% of *Salmonella* strains recovered from fattening pigs and 0.6% of *Salmonella* spp. from fattening pig carcasses, which belonged to different serovars (EFSA – European Food Safety Authority and ECDC – European Centre for Disease Prevention and Control 2019). In 2017, resistances to colistin were reported in 4.7% of all human *Salmonella* isolates with 88.9% of the resistant isolates belonging to either *S*. Enteritidis or *S*. Dublin (EFSA – European Food Safety Authority and ECDC – European Centre for Disease Prevention and Control 2019). Only seven EU member states reported data regarding colistin-resistant *Salmonella* strains, six of which detected colistin resistance, suggesting an underestimation of the actual resistance levels. ST32 is the most common sequence type among serovar Infantis, which is an increasingly important avian serovar. ST32 isolates from poultry farms in Serbia were found to harbor chromosomal colistin resistance (Jovcic *et al*. 2020). Additionally, *S*. Newport is an emerging serovar in human infections and chromosomal colistin resistance has been found in isolates from Europe and Asia (Olaitan *et al*. 2015; Jajere, 2019; Elbediwi *et al*. 2020). Chromosomal colistin resistance was also reported in further *S. enterica* isolated from human and animal samples from Asia and Europe (Fig. 5 and Table S3, Supporting Information; Luo *et al*. 2020).

#### *Enterobacter* spp.

*Enterobacter* spp. are part of the animal and human gut microbiome but also emerged as opportunistic human pathogens causing bacteremia, respiratory, urinary and gastrointestinal infections (Sanders and Sanders, 1997). Several studies observed that the prevalence of colistin resistance among human clinical *Enterobacterales* is higher in *Enterobacter* spp. than *E. coli* and *Klebsiella* spp. and was 0.7% in a global surveillance program, 1.5% in Tunisia, 4.2% in Spain, 4–20% in UK, 27.2% in Taiwan for *Enterobacter* species (Fig. 2; Maalej *et al*. 2012; Bradford *et al*. 2016; Prim *et al*. 2017; Jean *et al*. 2018; Mushtaq *et al*. 2020). Among CRE isolates, the prevalence of colistin resistance was as high as 54.1% for *Enterobacter* (Teo *et al*. 2019). The most prevalent colistin-resistant species detected were *E. asburiae* and *E. cloacae* but also found in *E. aerogenes*, *E. bugandensis* and other species (Bradford *et al*. 2016; Mushtaq *et al*. 2020; ATLAS database, https://atlas-surveillance.com/, accessed June 2021). A total of two major sequence types of carbapenem-resistant *E. cloacae* complex ST171 and ST78 have been described as epidemic, but chromosomal colistin resistance has been observed only in an ST78 isolate from Asia (Gomez-Simmonds *et al*. 2018; Teo *et al*. 2019). So far, there is no accumulation of chromosomal mutations in a particular sequence type and different sequence types from human clinical samples have been described from Europe and Asia (Fig. 5 and Table S3, Supporting Information; Majewski *et al*. 2014; Teo *et al*. 2019; Wand and Sutton, 2020). Worryingly, *Enterobacter* spp. exhibit the phenomenon of heteroresistance, which poses a significant problem for antimicrobial susceptibility testing in clinical settings (Hong, Lee and Ko 2018). Colistin heteroresistance can cause the 'skip well' phenomenon when using the BMD method for susceptibility testing, which is characterized by no bacterial growth at a certain antibiotic concentration, but growth takes place at higher antibiotic concentration (Landman, Salamera and Quale 2013). As a results, colistin heteroresistance may lead to treatment failure in clinical settings and may explain the high prevalence of colistin resistance among *Enterobacter* spp. (Fig. 2; Band *et al*. 2016). Interestingly, heteroresistance is observed more frequently in isolates belonging to a particular species or *E. cloacae* complex (Guerin *et al*. 2016).

### CONCLUSION

The global prevalence of colistin resistance in *Enterobacterales* and the significance of chromosomal mutations in mediating this resistance was assessed in the present review. To our knowledge, this study represents a critical comprehensive review on colistin-resistant *Enterobacterales*, including a comparison of their dissemination among European countries in the veterinary and human medicine, highlighting the role of mutational changes in chromosomal encoded genes. In the last 5 years, studies on colistin resistance comprised mainly the distribution of *mcr*-genes. However, resistance mechanisms seem to be more complex than previously thought. The genetic background of the bacterial species and the presence of supporting factors might play an important role and some enterobacterial genera (for example *Enterobacter* spp.) might contribute more than others to the development of colistin-resistance in *Enterobacterales*. Therefore, the impact of chromosomal mutations and their rate of emergence should not be overlooked. Our goal, with presenting these data, is to obtain a better understanding on the molecular basis of colistin resistance, which is necessary to be able to comprehend the development and spread of resistant isolates within the animal and human community.

Overall, the molecular basis of colistin resistance in *Enterobacterales* is very complex and not yet fully understood, whereby further clarification is urgently needed due to the increasing use of colistin as last-line antimicrobial in the clinic. Those findings highlight the need for routine WGS to define whether AMR is based on transfer of resistance determinants between different strains, or even species, or caused by spread of resistant strains. The genotypic results should be combined with experimental functional studies to understand the principles of colistin resistance. More importantly, the information should be linked with epidemiological data on AMR obtained from monitoring programs of the veterinary sector and human medicine. There is an urgent need for improved monitoring programs with real-time data reporting, especially for resistance towards those antimicrobials, which are used as last-line option for the treatment of serious infections in humans.

### ACKNOWLEDGMENTS

We want to thank all the scientists without their dedicated work this review would not have been possible. We also would like to thank the editor and the anonymous reviewers for their critical reading, thoughtful comments and constructive suggestions. Background world maps shown in Figure 2 and 5 were provided by Esri (https://www.arcgis.com/apps/mapviewer/index.html?webmap=8b3d38c0819547faa83f7b7aca80bd76).

## Supplementary Material

fuab049_Supplemental_Files
