# Supplementary material for: Global colistin use: a review of the emergence of resistant Enterobacterales and the impact on their genetic basis
Source: FEMS Microbiol Rev. 2021 Oct 6;46(1):fuab049. doi: 10.1093/femsre/fuab049 (PMC8829026; doi:10.1093/femsre/fuab049)
Supplement: fuab049_Supplemental_Files [file fuab049_supplemental_files.zip › Supplemental_Material_FEMSRE-21-03-0023.docx]

**Supplemental Material**

**Global colistin use: A review of the emergence of resistant *Enterobacterales* and the impact on their genetic basis**

Ulrike Binsker^1,*^, Annemarie Käsbohrer^1,2^, Jens A. Hammerl^1^

^1^ Department Biological Safety, German Federal Institute for Risk Assessment, Berlin, Germany

^2^ Department for Farm Animals and Veterinary Public Health, Institute of Veterinary Public Health, University of Veterinary Medicine Vienna, Vienna, Austria

*** Corresponding Author**

Dr. Ulrike Binsker

Address: Unit Epidemiology, Zoonoses and Antimicrobial Resistance, Department Biological Safety, German Federal Institute for Risk Assessment, Diedersdorfer Weg 1, 12277 Berlin, Germany

E-mail: Ulrike.Binsker@bfr.bund.de

Telephone: +49 30 18412 24340

**Brief overview about plasmid-encoded genes mediating colistin resistance**

In 2015, an unexpected increase of colistin-resistant *E. coli* retrieved from chicken and pigs in China was observed. Subsequent plasmid analysis of resistant isolates revealed the presence of a phosphoethanolamine transferase gene (*mcr-1*; mobile colistin resistance gene 1) on a transferrable plasmid [1]. The encoding enzyme is responsible for the transfer of pEtN to lipid A thereby mediating colistin resistance. Further investigation uncovered that the *mcr-1* gene was present in *E. coli* since 2011 and it has spread in isolates from livestock, raw meat products and even humans [1].

Thereafter, whole genome sequence (WGS) analysis throughout the world showed that the *mcr-1* gene was present in different continents [2]. Until 2018, the *mcr*-genes have been detected in 47 countries with a global frequency of 4.7% [3]. In Europe, the *mcr-1* gene has been present since 2004 where it has been found in *E. coli* from diseased cattle [4]. Additional species of the *Enterobacterales* family retrieved from livestock, vegetables, water, and more worryingly, MDR isolates from hospitalized patients, carry the *mcr-1* gene (Table 10). To date, ten different *mcr*-gene alleles (*mcr-2* [5], *mcr-3* [6], *mcr-4* [7], *mcr-5* [8] *mcr-6* [9], *mcr-7* [10], *mcr-8* [11], *mcr-9* [12], *mcr-10* [13]) have been characterized in a vast number of plasmid reservoirs. Interestingly, several variants have been described for *mcr*-*1* to *mcr*-*9*, of which *mcr*-*1* and *mcr*-*3* genes comprise the largest groups. After *mcr*-*1*, *mcr*-*9* is the most widespread plasmid-located colistin gene, which was found across six continents [2]. However, multiple studies report conflicting results whether *mcr*-*9* confers colistin resistance. For example, *Salmonella* and *E. coli* isolates from retail chicken meat in the US and different *Enterobacterales* strains isolated from horses in Sweden harbor the *mcr*-*9* gene but lack a colistin-resistant phenotype [14]. Discrepancies among the studies seem to depend on the species and isolate collection tested. The genes *mcr*-*2* to *mcr*-*7* are disseminated across European countries although *mcr*-*6* and *mcr*-*7* have been found only in United Kingdom and Germany, respectively [2]. Interestingly, co-occurrence of *mcr-1* with either *mcr-1*, *mcr-3*, *mcr-4*, or *mcr-5,* and *mcr-4* in combination with *mcr-5,* has been described in *E. coli* isolated from food-animals and companion animals in Spain, Japan, and China [15-22]. Furthermore, clinical *E. coli* and *K. pneumoniae* isolates from China, New Zealand, and Laos have been found to carry two *mcr*-genes [23-26]. The *mcr*-genes were located on the same plasmid, different plasmids or one variant was inserted into the chromosome [19-24]. Overall, the *mcr*-*1* gene is considered the most important due to its global distribution and its presence in a large number of different plasmids. Replicon types including IncX4, Incl2, IncHI1, IncHI2, IncFI, IncFII, IncP, and IncK types have been found to carry *mcr-1*, of which large conjugative plasmids, such as IncX4, IncI2, and IncHI2, are likely to be the most effective for its dissemination [22, 27-31]. The other *mcr*-genes, on the other hand, occur so far only locally and are rather associated with specific factors, such as species or plasmid replicon type. For example, *mcr-2* has been associated with the IncX4 replicon type, *mcr-3* is predominantly found on IncHI2 and IncP types, whereas *mcr-4* and *mcr-5* are harboured by non-conjugative ColE-like replicon plasmids [8, 19, 30-38].

**Table S1. Identification of *mcr*-positive** *Enterobacterales* **in European countries.**

| **Country** | **Date of Isolation** | **Source of isolate** | **Origin /**  **Travel history** | **Number of total samples**  **(*mcr*-positive) % positive** | *Enterobacterales* **(ST)** | ***mcr allele*** | ***mcr*-associated**  **plasmid replicon** | **Reference** |
| --- | --- | --- | --- | --- | --- | --- | --- | --- |
| Belgium | 2011-2012 | diarrhea in calves and piglets | ns | 105 (13) 12% | *E. coli* ST10, ST90, ST100, novel STs | *mcr-1* | IncP, IncFII [39] | [40] |
|  | 2012-2015  *2012 first mcr-positive isolate* | pork carcasses, cut poultry meat | ns | 105 (3) 2.9% | *Salmonella* ST34, 40, 3663 | *mcr-1, mcr-2* | IncX4 | [41] |
|  | 2016 | diarrhea in calves and piglets | ns | 10 (3) 30% | *E. coli* ST10 | *mcr-2* | IncX4 | [5] |
| Denmark | 2012-2015 | human blood,  chicken meat | ns | 914 (5 isolates from chicken meat, 1 human isolate) 0.7% | *E. coli* | *mcr-1* | IncI2, IncX4 | [42] |
|  | 2009–2017  *2009 first mcr-positive isolate* | human stool, blood, urine | Thailand, Vietnam,  unknown | NA (10) | *Salmonella* ST34 | *mcr-1, mcr-3* | IncHI2A, IncHI2, IncN, TrfA, IncQ1, ColRNAI, IncA/C2, IncFII, IncX1, IncFIC(FII), IncI2 | [43] |
| Estonia | 2011-2014  *2013 first mcr-positive isolate* | human, animals, environment | ns | 347 (3 isolates from pig slurry) 0.9% | *E. coli* | *mcr-1* | IncX4 | [44] |
| Europe-wide | 2002-2014  *2008 first mcr-positive isolate* | colon or cecal isolates from beef cattle, slaughter pigs, and broiler chickens | ns | 11 977 (68 E. coli,  2 Salmonella) 0.6% | *E. coli*, *Salmonella* | *mcr-1* | ns | [45] |
|  | 2004-2014  ***2004 first*** ***mcr-positive isolate from Italy*** | diseased cattle and pigs | ns | 292 (42 *E. coli*,  3 *Salmonella)* 15% | *E. coli* ST10*,* ST100, ST1615, ST410, ST167, ST1291, ST101, ST3088, ST760, ST20, ST162, ST5614, ST641, ST1011, ST624, ST1, ST88,  *Salmonella* | *mcr-1* | ns | [4] |
|  | 2013-2014 | human isolates | ns | 1717 (28) 1.6% | *K. pneumoniae* ST274, ST147, ST461, ST15, ST16, ST416, ST1890, ST37, ST1942 | *mcr-9* | ns | [46] |
| France | 2005-2014  *2005 first mcr-positive isolate* | feces of diarrheic veal calves | ns | 517 (106) 21% | *E. coli* | *mcr-1* | IncHI2 | [47] |
|  | 2007-2014 | broilers, pigs, and turkeys at slaughter | ns | 1450 (23) 1.6% | *E. coli* | *mcr-1* | ns | [48] |
|  | 2012-2013 | isolates from French agricultural food sector | ns | 8684 (4) 0.05% | *Salmonella* | *mcr-1* | IncP, IncX4 | [49] |
| Germany | 2009-2011  *2010 first mcr-positive isolate* | different sources (human, animal, and environment) | ns | 577 (4) 0.7% | *E. coli* | *mcr-1* | IncHI2, IncX4 | [50] |
|  | 2010-2015 | fecal and food samples of animal origin | ns | 10 600 (402) 3.8% | *E. coli* | *mcr-1* | ns | [51] |
|  | 2010-2017 | food-producing animals and food products | ns | 19 216 (3) 0.02% | *E. coli* | *mcr-5, mcr-5.2* | ColE, unknown incompatibility type | [52] |
|  | 2011–2012 | farm boot, manure, barn flies and barn dog feces | ns | 35 (7) 0.2% | *E. coli* ST10, ST1140, ST5281, ST1011, ST342 | *mcr-1* | IncX4 | [53] |
|  | 2011-2016 | livestock and food samples | ns | 32 (14) 44% | *S. enterica* Paratyphi B | *mcr-5* | ColE-type plasmid | [8] |
|  | 2011-2017 | pigs and meat | ns | 315 (8) 2.5% | *S.* Typhimurium ST34 | *mcr-5* | ColE-like, IncX1 | [54] |
|  | 2017 | hospitalized patients | ns | 1 217 (7) 0.6% | *E. coli* ST7056, ST8154, ST189, ST10, ST48, ST219, ST7329 | *mcr-1* | IncX4, IncHI2, IncI2 | [55] |
| Great Britain | 2012-2015  *2012 first mcr-positive isolate* | human and food isolates | countries in Asia, Africa,  meat imported from Europe | >24  000 (13 isolates from 12 humans, 2 isolates from poultry meat) 0.06% | *Enterobacterales* | *mcr-1* | IncI2, IncHI2, IncX4 | [28] |
|  | 2014-2015 | pigs | ns | 3 (3) | *Salmonella, E. coli* | *mcr-1* | repB, Incl2 | [56] |
|  | 2014-2017 | human, food, animal and environmental isolates | travel to South East Asia | 33 205 (52) 0.2% | *S. enterica* | *mcr-1, mcr-3*  *mcr-5* | IncX4, IncI2, IncHI2, IncHI1, IncA/C2, IncHI2A/IncY  chromosome | [57] |
| Italy | 2001-2017 | broilers, broiler meat samples | ns | 324 (4) 1.2% | *S.* Infantis ST32 | *mcr-1* | IncX4 | [58] |
|  | 2012-2015  *2013 first mcr-positive isolate* | hospitalized patients | ns | 44 530 (8) 0.02% | *E. coli* ST10, ST354, ST131, ST602, ST95, ST648, ST804, ST117 | *mcr-1* | ns | [59] |
|  | 2012-2015 | human, animal, food, and environmental sources | ns | 4473 (10 humans,  15 veterinary sources) 0.6% | *Salmonella* | *mcr-1* | ns | [60] |
|  | 2013 | pig at slaughter | ns | NA (1) | *S.* Typhimurium ST34 | *mcr-4* | ColE10 | [7] |
|  | 2014 | rectal swabs | ns | NA (1) | *K. pneumoniae* ST512 | *mcr-1.2* | IncX4 | [61] |
|  | 2014–2015 | well/stream | ns | 132 (3 *E. coli*,  1 *K. pneumoniae*) 3.0% | *E. coli*, *K. pneumoniae* | *mcr-1,*  *mcr-1.2* | IncX4 | [62] |
|  | 2014-2015 | food-producing animals | ns | 1775 (92 *E. coli*,  12 *Salmonella*) 5.9% | *E. coli*, *Salmonella* | *mcr-1 - mcr5* | IncX4 | [63] |
|  | 2016 | human fecal samples | ns | 2 (2) | *S.* Typhimurium | *mcr-4.2* | ns | [64] |
|  | 2016–2017 | hospital surfaces | ns | 300 (25) 8.3% | *Enterobacterales* | *mcr-1* | ns | [65] |
|  | 2016–2017 | hospitalized patients with bacteremia, blood and urine samples | not travelled abroad | 3 (3) | *E. coli* ST 131, 3941, 1851 | *mcr-1* | ns | [66] |
| Lithuania | 2016 | rectal swabs/feces of *Larus argentatus* (migratory bird) | ns | 117 (1) 0.85% | *E. coli* | *mcr-1* | IncI | [67] |
| Netherlands | 2009-2014  *2009 first mcr-positive isolate* | retail chicken meat | ns | 2471 (3) 0.12% | *E. coli* ST2079, ST117 | *mcr-1* | ns | [68] |
|  | 2010-2012 | fecal samples of healthy Dutch travelers | South(-east) Asia, southern Africa | 122 (6) 4.9% | *E. coli* ST1011, ST744, ST80 | *mcr-1* | ns | [69] |
|  | 2010-2015 | hospitalized patients | ns | 18 (3) 17% | *E. coli* | *mcr-1* | ns | [70] |
|  | 2010-2015 | fecal samples of veal calves, broilers, turkey, broiler meat | ns | 39 (39) | *E. coli*, *Salmonella* | *mcr-1* | IncHI2, IncX4,  2 chromosomal *mcr*-1 | [71] |
| Norway | 2010 | water from public beach | ns | 82 (2) 2.4% | *E. coli* ST10 (A and B1) | *mcr-1* | ns | [72] |
|  | 2016 | imported sea food, dog food | scampi from Bangladesh, dog food from UK | 2 (2) | *E. coli* ST48, ST3014 | *mcr-1* | ns | [73] |
| Poland | 2015 | hospitalized patient | none | 1 (1) | *E. coli* ST617 | *mcr-1* | IncI2 | [74] |
| Portugal | 2002-2015  *2011 first mcr-positive isolate* | human feces/blood, pig at slaughter, pork food product | ns | 1010 (11) 1.1% | *Salmonella* | *mcr-1* | IncX4, IncHI2 | [75] |
|  | 2011-2012 | animal food products | ns | 258 (4) 1.6% | *S.* Typhimurium | *mcr-1* | IncHI2 | [76] |
|  | 2013–2014 | vegetables, fruits | ns | 138 (1) 0.72% | *E. coli* ST1716 | *mcr-1* | ns | [77] |
| Spain | 2009-2014 | feces from turkey and swine, swine lymph nodes | ns | ns (9) | *E. coli*, *Salmonella* | *mcr-1* | ns | [78] |
|  | 2012-2015  *2012 first mcr-positive isolate* | clinical isolates | ns | 10 011 (15) 0.15% | *E. coli* | *mcr-1* | ns | [79] |
|  | 2012–2016 | mussels | ns | 19 (1) 5.3% | *S.* Risen ST469 (*invA*) | *mcr-1* | ns | [80] |
|  | 2013 | sewage water | ns | 90 (30) 33% | *E. coli*, *K. pneumoniae* | *mcr-1* | Inc2 | [81] |
|  | 2015- 2016 | cloacal swabs of a wild bird (black vulture) | ns | 94 (1) 1.1% | *E. coli* ST162 | *mcr-1* | ns | [82] |
| Switzerland | 2012 -2014  *2012 first mcr-positive isolate* | lake/river water and vegetables | imported vegetable from Thailand and Vietnam | 74 water (1) 1.4%  60 vegetable (2) 3.3% | 1 *E. coli* ST359 from water,  2 *E. coli* ST167, ST4683  from vegetable | *mcr-1* | ns | [83] |
|  | 2014-2016 | human feces, chicken meat | meat from Germany | 9 (9) | *E. coli,* human: ST10, ST5  meat: ST38, ST58, ST1775, ST226, ST1049 | *mcr-1* | IncHI2, IncX4, IncI2, IncK2, chromosome | [84] |
|  | 2015 | chicken meat | Germany | 1 (1) | *E. coli* | *mcr-1* | chromosome | [85] |
|  | 2015 | blood from bacteremia patients | ns | 2 (2) | *E. coli* | *mcr-1* | IncFIB | [86] |
|  | 2016 | urinary isolates from humans | ns | 2049 (1) 0.05% | *E. coli* ST428 | *mcr-1* | ns | [87] |
|  | 2017 | human blood sample | unknown | 1 (1) | *S. enterica* 4,5,12:i: | *mcr-1* | IncX4 | [88] |

ns: not specified

**Table S2: Global colistin use in the veterinary and human medicine**

| **Continent** | **Colistin use in veterinary medicine** | **Colistin use in human medicine** |
| --- | --- | --- |
| **Europe** | - 1950s, approved at national level - 2006, ban of colistin as growth promoter [89] - 2014, introduction of mandatory susceptibility testing to colistin for bacteria isolated from food-producing animals (Regulation 2013/652/EU) [90] | - 2016, polymyxins classified as critically important antimicrobial with highest priority (HPCIA) for human medicine by WHO [91] |
|  | - 2017, implementation of One Health approach to fight AMR in animal and human sector and to prevent transmission of resistant bacteria [92] | |
|  | - 2018, polymyxins classified into category “Veterinary Highly Important Antimicrobial Agents” by OIE [93] |  |
|  | - 2019, polymyxins classified into category B “Restrict” of antibiotics used in veterinary medicine by EMA [94] |  |
| **Asia** |  |  |
| China | - 2016, ban of colistin as growth promoter [95] | - 2017, approval of polymyxin B [96] - 2018, approval of colistin [96] |
| India | - 2019, ban of colistin as growth promoter and therapeutic agent (section 26A of Drugs and Cosmetics Act, 1940) | - 1962, approval of colistin - 1977, approval of polymyxin B   (https://cdscoonline.gov.in/CDSCO/Drugs) |
| Japan | - 2018, ban of colistin as growth promoter - 2018, therapeutic colistin was moved from first to second-choice drug by the Ministry of Agriculture, Forestry, and Fisheries of Japan [97] | - 2015, approval of colistin [98] |
| **Australia** | - Polymyxin B but not colistin was approved for the use in livestock animals [99] | - 1991, approval of colistin (https://www.tga.gov.au/) |
| **North America** |  |  |
| USA | - 1998, Colistin has been approved but never been marketed - 2009, Polymyxin B is approved and used   (https://animaldrugsatfda.fda.gov/ adafda/views/#/search) | - 1970, approval of colistin - 1994, approval of polymyxin B   (https://www.accessdata.fda.gov/scripts/ cder/daf/index.cfm) |
| Canada | - 1989, approval for polymyxin B   (https://health-products.canada.ca/dpd-bdpp/) | - 1963, approval of polymyxin B - 1974, approval of colistin   (https://health-products.canada.ca/dpd-bdpp/) |
| **Central and South America** |  |  |
| Argentina | - 2015, ban of colistin as growth promoter - 2019, ban of colistin in veterinary medicine [100] | - Colistin is approved [101] |
| Bolivia | - Status 2021: use of colistin as therapeutic agent |  |
| Brazil | - 2016, ban of colistin as growth promoter - Status 2021: use of colistin as therapeutic agent |  |
| Chile | - Status 2021: use of colistin as therapeutic agent |  |
| Colombia | - Status 2021: use of colistin as therapeutic agent |  |
| Costa Rica | - Status 2021: use of colistin not permitted |  |
| Cuba | - Status 2021: use of colistin as therapeutic agent |  |
| Ecuador | - Status 2021: use of colistin as therapeutic agent |  |
| Mexico | - Status 2021: use of colistin as therapeutic agent |  |
| Nicaragua | - Status 2021: use of colistin not permitted |  |
| Paraguay | - 2019, ban of colistin as growth promoter and therapeutic agent |  |
| Peru | - Status 2021: use of colistin not permitted |  |
| Uruguay | - Status 2021: use of colistin as therapeutic agent |  |
| **Africa** |  |  |
| South Africa | - Colistin is approved, its use is not recommended but possible under certain circumstances [102] - 2016: ban of colistin as growth promoter | - Colistin is not registered and only available in exceptional circumstances via the Medicines and Related Substances Act,   through the Section 21 application process to the Medicines Control Council (MCC) [103, 104] |
| Tunisia |  | - Colistin is approved [105] |

**References**

1. Liu, Y.Y., Y. Wang, T.R. Walsh, et al., *Emergence of plasmid-mediated colistin resistance mechanism MCR-1 in animals and human beings in China: a microbiological and molecular biological study.* Lancet Infectious Diseases, 2016. **16**(2): p. 161-168.

2. Ling, Z., W. Yin, Z. Shen, et al., *Epidemiology of mobile colistin resistance genes mcr-1 to mcr-9.* J Antimicrob Chemother, 2020. **75**(11): p. 3087-3095.

3. Elbediwi, M., Y. Li, N. Paudyal, et al., *Global Burden of Colistin-Resistant Bacteria: Mobilized Colistin Resistance Genes Study (1980-2018).* Microorganisms, 2019. **7**(10).

4. El Garch, F., M. Sauget, D. Hocquet, et al., *mcr-1 is borne by highly diverse Escherichia coli isolates since 2004 in food-producing animals in Europe.* Clinical Microbiology and Infection, 2017. **23**(1).

5. Xavier, B.B., C. Lammens, R. Ruhal, et al., *Identification of a novel plasmid-mediated colistin-resistance gene, mcr-2, in Escherichia coli, Belgium, June 2016.* Eurosurveillance, 2016. **21**(27): p. 8-13.

6. Yin, W.J., H. Li, Y.B. Shen, et al., *Novel Plasmid-Mediated Colistin Resistance Gene mcr-3 in Escherichia coli.* Mbio, 2017. **8**(3).

7. Carattoli, A., L. Villa, C. Feudi, et al., *Novel plasmid-mediated colistin resistance mcr-4 gene in Salmonella and Escherichia coli, Italy 2013, Spain and Belgium, 2015 to 2016.* Eurosurveillance, 2017. **22**(31): p. 18-22.

8. Borowiak, M., J. Fischer, J.A. Hammerl, et al., *Identification of a novel transposon-associated phosphoethanolamine transferase gene, mcr-5, conferring colistin resistance in d-tartrate fermenting Salmonella enterica subsp enterica serovar Paratyphi B.* Journal of Antimicrobial Chemotherapy, 2017. **72**(12): p. 3317-3324.

9. AbuOun, M., E.J. Stubberfield, N.A. Duggett, et al., *mcr-1 and mcr-2 (mcr-6.1) variant genes identified in Moraxella species isolated from pigs in Great Britain from 2014 to 2015 (vol 72, pg 2745, 2018).* Journal of Antimicrobial Chemotherapy, 2018. **73**(10): p. 2904-2904.

10. Yang, Y.Q., Y.X. Li, C.W. Lei, et al., *Novel plasmid-mediated colistin resistance gene mcr-7.1 in Klebsiella pneumoniae.* Journal of Antimicrobial Chemotherapy, 2018. **73**(7): p. 1791-1795.

11. Wang, X.M., Y. Wang, Y. Zhou, et al., *Emergence of a novel mobile colistin resistance gene, mcr-8, in NDM-producing Klebsiella pneumoniae.* Emerging Microbes & Infections, 2018. **7**.

12. Carroll, L.M., A. Gaballa, C. Guldimann, et al., *Identification of Novel Mobilized Colistin Resistance Gene mcr-9 in a Multidrug-Resistant, Colistin-Susceptible Salmonella enterica Serotype Typhimurium Isolate.* Mbio, 2019. **10**(3).

13. Wang, C.C., Y. Feng, L.N. Liu, et al., *Identification of novel mobile colistin resistance gene mcr-10.* Emerging Microbes & Infections, 2020. **9**(1): p. 508-516.

14. Tyson, G.H., C. Li, C.H. Hsu, et al., *The mcr-9 Gene of Salmonella and Escherichia coli Is Not Associated with Colistin Resistance in the United States.* Antimicrob Agents Chemother, 2020. **64**(8).

15. Hernandez, M., M.R. Iglesias, D. Rodriguez-Lazaro, et al., *Co-occurrence of colistin-resistance genes mcr-1 and mcr-3 among multidrug-resistant Escherichia coli isolated from cattle, Spain, September 2015.* Euro Surveill, 2017. **22**(31).

16. Fukuda, A., T. Sato, M. Shinagawa, et al., *High prevalence of mcr-1, mcr-3 and mcr-5 in Escherichia coli derived from diseased pigs in Japan.* Int J Antimicrob Agents, 2018. **51**(1): p. 163-164.

17. Rebelo, A.R., V. Bortolaia, J.S. Kjeldgaard, et al., *Multiplex PCR for detection of plasmid-mediated colistin resistance determinants, mcr-1, mcr-2, mcr-3, mcr-4 and mcr-5 for surveillance purposes.* Eurosurveillance, 2018. **23**(6): p. 29-39.

18. Garcia, V., I. Garcia-Menino, A. Mora, et al., *Co-occurrence of mcr-1, mcr-4 and mcr-5 genes in multidrug-resistant ST10 Enterotoxigenic and Shiga toxin-producing Escherichia coli in Spain (2006-2017).* Int J Antimicrob Agents, 2018. **52**(1): p. 104-108.

19. Du, C.T., Y.Y. Feng, G.Z. Wang, et al., *Co-Occurrence of the mcr-1.1 and mcr-3.7 Genes in a Multidrug-Resistant Escherichia coli Isolate from China.* Infection and Drug Resistance, 2020. **13**: p. 3649-3655.

20. Sun, J., X.P. Li, L.X. Fang, et al., *Co-occurrence of mcr-1 in the chromosome and on an IncHI2 plasmid: persistence of colistin resistance in Escherichia coli.* International Journal of Antimicrobial Agents, 2018. **51**(6): p. 842-847.

21. Li, R.C., P. Zhang, X.R. Yang, et al., *Identification of a novel hybrid plasmid coproducing MCR-1 and MCR-3 variant from an Escherichia coli strain.* Journal of Antimicrobial Chemotherapy, 2019. **74**(6): p. 1517-1520.

22. Wang, Q.J., J. Sun, J. Li, et al., *Expanding landscapes of the diversified mcr-1-bearing plasmid reservoirs.* Microbiome, 2017. **5**.

23. Liu, L., Y. Feng, X. Zhang, et al., *New Variant of mcr-3 in an Extensively Drug-Resistant Escherichia coli Clinical Isolate Carrying mcr-1 and blaNDM-5.* Antimicrob Agents Chemother, 2017. **61**(12).

24. Creighton, J., T. Anderson, J. Howard, et al., *Co-occurrence of mcr-1 and mcr-3 genes in a single Escherichia coli in New Zealand.* Journal of Antimicrobial Chemotherapy, 2019. **74**(10): p. 3113-3116.

25. Hala, S., C.P. Antony, A.A. Momin, et al., *Co-occurrence of mcr-1 and mcr-8 genes in multi-drug-resistant Klebsiella pneumoniae from a 2015 clinical isolate.* Int J Antimicrob Agents, 2021. **57**(3): p. 106303.

26. Hadjadj, L., S.A. Baron, A.O. Olaitan, et al., *Co-occurrence of Variants of mcr-3 and mcr-8 Genes in a Klebsiella pneumoniae Isolate From Laos.* Frontiers in Microbiology, 2019. **10**.

27. Poirel, L., A. Jayol, and P. Nordmann, *Polymyxins: Antibacterial Activity, Susceptibility Testing, and Resistance Mechanisms Encoded by Plasmids or Chromosomes.* Clin Microbiol Rev, 2017. **30**(2): p. 557-596.

28. Doumith, M., G. Godbole, P. Ashton, et al., *Detection of the plasmid-mediated mcr-1 gene conferring colistin resistance in human and food isolates of Salmonella enterica and Escherichia coli in England and Wales.* Journal of Antimicrobial Chemotherapy, 2016. **71**(8): p. 2300-2305.

29. Manageiro, V., L. Clemente, R. Romao, et al., *IncX4 Plasmid Carrying the New mcr-1.9 Gene Variant in a CTX-M-8-Producing Escherichia coli Isolate Recovered From Swine.* Frontiers in Microbiology, 2019. **10**.

30. Garcia-Menino, I., D. Diaz-Jimenez, V. Garcia, et al., *Genomic Characterization of Prevalent mcr-1, mcr-4, and mcr-5 Escherichia coli Within Swine Enteric Colibacillosis in Spain.* Frontiers in Microbiology, 2019. **10**.

31. Sun, J., H.M. Zhang, Y.H. Liu, et al., *Towards Understanding MCR-like Colistin Resistance.* Trends in Microbiology, 2018. **26**(9): p. 794-808.

32. Sun, J., Y.C. Xu, R.S. Gao, et al., *Deciphering MCR-2 Colistin Resistance.* Mbio, 2017. **8**(3).

33. Long, H., Y. Feng, K. Ma, et al., *The co-transfer of plasmid-borne colistin-resistant genes mcr-1 and mcr-3.5, the carbapenemase gene blaNDM-5 and the 16S methylase gene rmtB from Escherichia coli.* Sci Rep, 2019. **9**(1): p. 696.

34. Xu, Y.C., L.L. Zhong, S. Srinivas, et al., *Spread of MCR-3 Colistin Resistance in China: An Epidemiological, Genomic and Mechanistic Study.* Ebiomedicine, 2018. **34**: p. 139-157.

35. Wang, Z., Y. Fu, S. Schwarz, et al., *Genetic environment of colistin resistance genes mcr-1 and mcr-3 in Escherichia coli from one pig farm in China.* Vet Microbiol, 2019. **230**: p. 56-61.

36. Mechesso, A.F., D.C. Moon, H.Y. Kang, et al., *Emergence of mcr-3 carrying Escherichia coli in Diseased Pigs in South Korea.* Microorganisms, 2020. **8**(10).

37. Xiang, R., B.H. Liu, A.Y. Zhang, et al., *Colocation of the Polymyxin Resistance Gene mcr-1 and a Variant of mcr-3 on a Plasmid in an Escherichia coli Isolate from a Chicken Farm.* Antimicrob Agents Chemother, 2018. **62**(6).

38. Carattoli, A., E. Carretto, F. Brovarone, et al., *Comparative analysis of an mcr-4 Salmonella enterica subsp. enterica monophasic variant of human and animal origin.* J Antimicrob Chemother, 2018. **73**(12): p. 3332-3335.

39. Xavier, B.B., C. Lammens, P. Butaye, et al., *Complete sequence of an IncFII plasmid harbouring the colistin resistance gene mcr-1 isolated from Belgian pig farms.* Journal of Antimicrobial Chemotherapy, 2016. **71**(8): p. 2342-2344.

40. Malhotra-Kumar, S., B.B. Xavier, A.J. Das, et al., *Colistin resistance gene mcr-1 harboured on a multidrug resistant plasmid.* Lancet Infectious Diseases, 2016. **16**(3): p. 283-284.

41. Garcia-Graells, C., S.C.J. De Keersmaecker, K. Vanneste, et al., *Detection of Plasmid-Mediated Colistin Resistance, mcr-1 and mcr-2 Genes, in Salmonella spp. Isolated from Food at Retail in Belgium from 2012 to 2015.* Foodborne Pathogens and Disease, 2018. **15**(2): p. 114-117.

42. Hasman, H., A.M. Hammerum, F. Hansen, et al., *Detection of mcr-1 encoding plasmid-mediated colistin-resistant Escherichia coli isolates from human bloodstream infection and imported chicken meat, Denmark 2015.* Eurosurveillance, 2015. **20**(49): p. 2-6.

43. Litrup, E., K. Kiil, A.M. Hammerum, et al., *Plasmid-borne colistin resistance gene mcr-3 in Salmonella isolates from human infections, Denmark, 2009-17.* Eurosurveillance, 2017. **22**(31): p. 10-12.

44. Brauer, A., K. Telling, M. Laht, et al., *Plasmid with Colistin Resistance Gene mcr-1 in Extended-Spectrum-beta-Lactamase-Producing Escherichia coli Strains Isolated from Pig Slurry in Estonia.* Antimicrob Agents Chemother, 2016. **60**(11): p. 6933-6936.

45. El Garch, F., A. de Jong, X. Bertrand, et al., *mcr-1-like detection in commensal Escherichia coli and Salmonella spp. from food-producing animals at slaughter in Europe.* Veterinary Microbiology, 2018. **213**: p. 42-46.

46. Wang, Y.N., F. Liu, Y.F. Hu, et al., *Detection of mobile colistin resistance gene mcr-9 in carbapenem-resistant Klebsiella pneumoniae strains of human origin in Europe.* Journal of Infection, 2020. **80**(5): p. 588-590.

47. Haenni, M., L. Poirel, N. Kieffer, et al., *Co-occurrence of extended spectrum beta lactamase and MCR-1 encoding genes on plasmids.* Lancet Infect Dis, 2016. **16**(3): p. 281-2.

48. Perrin-Guyomard, A., M. Bruneau, P. Houee, et al., *Prevalence of mcr-1 in commensal Escherichia coli from French livestock, 2007 to 2014.* Eurosurveillance, 2016. **21**(6): p. 6-8.

49. Webb, H.E., S.A. Granier, M. Marault, et al., *Dissemination of the mcr-1 colistin resistance gene.* Lancet Infectious Diseases, 2016. **16**(2): p. 144-145.

50. Falgenhauer, L., S.E. Waezsada, Y. Yao, et al., *Colistin resistance gene mcr-1 in extended-spectrum beta-lactamase-producing and carbapenemase-producing Gram-negative bacteria in Germany.* Lancet Infect Dis, 2016. **16**(3): p. 282-3.

51. Irrgang, A., N. Roschanski, B.A. Tenhagen, et al., *Prevalence of mcr-1 in E. coli from Livestock and Food in Germany, 2010-2015.* Plos One, 2016. **11**(7).

52. Hammerl, J.A., M. Borowiak, S. Schmoger, et al., *mcr-5 and a novel mcr-5.2 variant in Escherichia coli isolates from food and food-producing animals, Germany, 2010 to 2017.* Journal of Antimicrobial Chemotherapy, 2018. **73**(5): p. 1433-1435.

53. Guenther, S., L. Falgenhauer, T. Semmler, et al., *Environmental emission of multiresistant Escherichia coli carrying the colistin resistance gene mcr-1 from German swine farms.* Journal of Antimicrobial Chemotherapy, 2017. **72**(5): p. 1289-1292.

54. Borowiak, M., J.A. Hammerl, C. Deneke, et al., *Characterization of mcr-5-Harboring Salmonella enterica subsp. enterica Serovar Typhimurium Isolates from Animal and Food Origin in Germany.* Antimicrobial Agents and Chemotherapy, 2019. **63**(6).

55. Bourrel, A.S., L. Poirel, G. Royer, et al., *Colistin resistance in Parisian inpatient faecal Escherichia coli as the result of two distinct evolutionary pathways.* Journal of Antimicrobial Chemotherapy, 2019. **74**(6): p. 1521-1530.

56. Anjum, M.F., N.A. Duggett, M. AbuOun, et al., *Colistin resistance in Salmonella and Escherichia coli isolates from a pig farm in Great Britain.* Journal of Antimicrobial Chemotherapy, 2016. **71**(8): p. 2306-2313.

57. Sia, C.M., D.R. Greig, M. Day, et al., *The characterization of mobile colistin resistance (mcr) genes among 33 000 Salmonella enterica genomes from routine public health surveillance in England.* Microbial Genomics, 2020. **6**(2).

58. Carfora, V., P. Alba, P. Leekitcharoenphon, et al., *Colistin Resistance Mediated by mcr-1 in ESBL-Producing, Multidrug Resistant Salmonella Infantis in Broiler Chicken Industry, Italy (2016-2017).* Frontiers in Microbiology, 2018. **9**.

59. Cannatelli, A., T. Giani, A. Antonelli, et al., *First Detection of the mcr-1 Colistin Resistance Gene in Escherichia coli in Italy.* Antimicrobial Agents and Chemotherapy, 2016. **60**(5): p. 3257-3258.

60. Carnevali, C., M. Morganti, E. Scaltriti, et al., *Occurrence of mcr-1 in Colistin-Resistant Salmonella enterica Isolates Recovered from Humans and Animals in Italy, 2012 to 2015.* Antimicrobial Agents and Chemotherapy, 2016. **60**(12): p. 7532-7534.

61. Di Pilato, V., F. Arena, C. Tascini, et al., *mcr-1.2, a New mcr Variant Carried on a Transferable Plasmid from a Colistin-Resistant KPC Carbapenemase-Producing Klebsiella pneumoniae Strain of Sequence Type 512.* Antimicrobial Agents and Chemotherapy, 2016. **60**(9): p. 5612-5615.

62. Caltagirone, M., E. Nucleo, M. Spalla, et al., *Occurrence of Extended Spectrum beta-Lactamases, KPC-Type, and MCR-1.2-Producing Enterobacteriaceae from Wells, River Water, and Wastewater Treatment Plants in Oltrepo Pavese Area, Northern Italy.* Front Microbiol, 2017. **8**: p. 2232.

63. Alba, P., P. Leekitcharoenphon, A. Franco, et al., *Molecular Epidemiology of mcr-Encoded Colistin Resistance in Enterobacteriaceae From Food-Producing Animals in Italy Revealed Through the EU Harmonized Antimicrobial Resistance Monitoring.* Frontiers in Microbiology, 2018. **9**.

64. Carretto, E., F. Brovarone, P. Nardini, et al., *Detection of mcr-4 positive Salmonella enterica serovar Typhimurium in clinical isolates of human origin, Italy, October to November 2016.* Eurosurveillance, 2018. **23**(2): p. 9-11.

65. Caselli, E., M. D'Accolti, I. Soffritti, et al., *Spread of mcr-1-Driven Colistin Resistance on Hospital Surfaces, Italy.* Emerging Infectious Diseases, 2018. **24**(9): p. 1752-1753.

66. Corbella, M., B. Mariani, C. Ferrari, et al., *Three cases of mcr-1-positive colistin-resistant Escherichia coli bloodstream infections in Italy, August 2016 to January 2017.* Eurosurveillance, 2017. **22**(16): p. 2-5.

67. Ruzauskas, M. and L. Vaskeviciute, *Detection of the mcr-1 gene in Escherichia coli prevalent in the migratory bird species Larus argentatus.* Journal of Antimicrobial Chemotherapy, 2016. **71**(8): p. 2333-2334.

68. Kluytmans-van den Bergh, M.F., P. Huizinga, M.J. Bonten, et al., *Presence of mcr-1-positive Enterobacteriaceae in retail chicken meat but not in humans in the Netherlands since 2009.* Eurosurveillance, 2016. **21**(9): p. 12-18.

69. von Wintersdorff, C.J.H., P.F.G. Wolffs, J.M. van Niekerk, et al., *Detection of the plasmid-mediated colistin-resistance gene mcr-1 in faecal metagenomes of Dutch travellers.* Journal of Antimicrobial Chemotherapy, 2016. **71**(12): p. 3416-3419.

70. Nijhuis, R.H.T., K.T. Veldman, J. Schelfaut, et al., *Detection of the plasmid-mediated colistin-resistance gene mcr-1 in clinical isolates and stool specimens obtained from hospitalized patients using a newly developed real-time PCR assay.* Journal of Antimicrobial Chemotherapy, 2016. **71**(8): p. 2344-2346.

71. Veldman, K., A. van Essen-Zandbergen, M. Rapallini, et al., *Location of colistin resistance gene mcr-1 in Enterobacteriaceae from livestock and meat.* Journal of Antimicrobial Chemotherapy, 2016. **71**(8): p. 2340-2342.

72. Jorgensen, S.B., A. Soraas, L.S. Arnesen, et al., *First environmental sample containing plasmid-mediated colistin-resistant ESBL-producing Escherichia coli detected in Norway.* Apmis, 2017. **125**(9): p. 822-825.

73. Slettemeas, J.S., A.M. Urdahl, S.S. Mo, et al., *Imported food and feed as contributors to the introduction of plasmid-mediated colistin-resistant Enterobacteriaceae to a 'low prevalence' country.* Journal of Antimicrobial Chemotherapy, 2017. **72**(9): p. 2675-2677.

74. Izdebski, R., A. Baraniak, K. Bojarska, et al., *Mobile MCR-1-associated resistance to colistin in Poland.* Journal of Antimicrobial Chemotherapy, 2016. **71**(8): p. 2331-2333.

75. Campos, J., L. Cristino, L. Peixe, et al., *MCR-1 in multidrug-resistant and copper-tolerant clinically relevant Salmonella 1,4,[5],12: i:- and S. Rissen clones in Portugal, 2011 to 2015.* Eurosurveillance, 2016. **21**(26): p. 2-6.

76. Figueiredo, R., R.M. Card, J. Nunez, et al., *Detection of an mcr-1-encoding plasmid mediating colistin resistance in Salmonella enterica from retail meat in Portugal.* Journal of Antimicrobial Chemotherapy, 2016. **71**(8): p. 2338-2340.

77. Jones-Dias, D., V. Manageiro, E. Ferreira, et al., *Architecture of Class 1, 2, and 3 Integrons from Gram Negative Bacteria Recovered among Fruits and Vegetables.* Frontiers in Microbiology, 2016. **7**.

78. Quesada, A., M. Ugarte-Ruiz, M.R. Iglesias, et al., *Detection of plasmid mediated colistin resistance (MCR-1) in Escherichia coli and Salmonella enterica isolated from poultry and swine in Spain.* Research in Veterinary Science, 2016. **105**: p. 134-135.

79. Prim, N., A. Rivera, J. Rodriguez-Navarro, et al., *Detection of mcr-1 colistin resistance gene in polyclonal Escherichia coli isolates in Barcelona, Spain, 2012 to 2015.* Eurosurveillance, 2016. **21**(13): p. 5-7.

80. Lozano-Leon, A., C. Garcia-Omil, J. Dalama, et al., *Detection of colistin resistance mcr-1 gene in Salmonella enterica serovar Rissen isolated from mussels, Spain, 2012 to 2016.* Eurosurveillance, 2019. **24**(16): p. 2-6.

81. Ovejero, C.M., J.F. Delgado-Blas, W. Calero-Caceres, et al., *Spread of mcr-1-carrying Enterobacteriaceae in sewage water from Spain.* Journal of Antimicrobial Chemotherapy, 2017. **72**(4): p. 1050-1053.

82. Oteo, J., A. Mencia, V. Bautista, et al., *Colonization with Enterobacteriaceae-Producing ESBLs, AmpCs, and OXA-48 in Wild Avian Species, Spain 2015-2016.* Microbial Drug Resistance, 2018. **24**(7): p. 932-938.

83. Zurfuh, K., L. Poirel, P. Nordmann, et al., *Occurrence of the Plasmid-Borne mcr-1 Colistin Resistance Gene in Extended-Spectrum-beta-Lactamase-Producing Enterobacteriaceae in River Water and Imported Vegetable Samples in Switzerland.* Antimicrob Agents Chemother, 2016. **60**(4): p. 2594-5.

84. Dona, V., O.J. Bernasconi, J. Pires, et al., *Heterogeneous Genetic Location of mcr-1 in Colistin-Resistant Escherichia coli Isolates from Humans and Retail Chicken Meat in Switzerland: Emergence of mcr-1-Carrying IncK2 Plasmids.* Antimicrobial Agents and Chemotherapy, 2017. **61**(11).

85. Zurfluh, K., T. Tasara, L. Poirel, et al., *Draft Genome Sequence of Escherichia coli S51, a Chicken Isolate Harboring a Chromosomally Encoded mcr-1 Gene.* Genome Announc, 2016. **4**(4).

86. Nordmann, P., R. Lienhard, N. Kieffer, et al., *Plasmid-Mediated Colistin-Resistant Escherichia coli in Bacteremia in Switzerland.* Clinical Infectious Diseases, 2016. **62**(10): p. 1322-1323.

87. Liassine, N., L. Assouvie, M.C. Descombes, et al., *Very low prevalence of MCR-1/MCR-2 plasmid-mediated colistin resistance in urinary tract Enterobacteriaceae in Switzerland.* International Journal of Infectious Diseases, 2016. **51**: p. 4-5.

88. Carroll, L.M., K. Zurfluh, H. Jang, et al., *First report of an mcr-1- harboring Salmonella enterica subsp enterica serotype 4,5,12: i:- strain isolated from blood of a patient in Switzerland.* International Journal of Antimicrobial Agents, 2018. **52**(5): p. 740-741.

89. European Commission, *Ban on antibiotics as growth promoters in animal feed enters into effect*. 2005 [cited 2020 8th December]; Available from: https://ec.europa.eu/commission/presscorner/detail/en/IP_05_1687.

90. European Commission, *Commission implementing decision 2013/652/EU of 12 November 2013 on the monitoring and reporting of antimicrobial resistance in zoonotic and commensal bacteria.* Official Journal of the European Union, 2013.

91. World Health Organization (WHO), *Critically important antimicrobials for human medicine, 6th revision. Geneva: World Health Organization.* 2019: p. 45.

92. European Commission, *A European One Health Action Plan against Antimicrobial Resistance (AMR).* 2017.

93. World Organisation for Animal Health (OIE), *OIE LIST OF ANTIMICROBIAL AGENTS OF VETERINARY IMPORTANCE.* 2018.

94. European Medicines Agency (EMA)*,Categorisation of antibiotics in the European Union.* 2019.

95. Walsh, T.R. and Y.N. Wu, *China bans colistin as a feed additive for animals.* Lancet Infectious Diseases, 2016. **16**(10): p. 1102-1103.

96. Zhang, R., Y.B. Shen, T.R. Walsh, et al., *Use of polymyxins in Chinese hospitals.* Lancet Infectious Diseases, 2020. **20**(10): p. 1125-1126.

97. Usui, M., Y. Nozawa, A. Fukuda, et al., *Decreased colistin resistance and mcr-1 prevalence in pig-derived Escherichia coli in Japan after banning colistin as a feed additive.* Journal of Global Antimicrobial Resistance, 2021. **24**: p. 383-386.

98. Hamada, Y., J. Hirai, H. Suematsu, et al., *Clinical experience with colistin in 9 Japanese patients with infection due to multi-drug resistance pathogens.* Jpn J Antibiot, 2016. **69**(5): p. 319-326.

99. Commonwealth of Australia, *Importance Ratings and Summary of Antibacterial Uses in Human and Animal Health in Australia.* 2018.

100. Argentina Ministry of Justice and Human Rights, *RESOL-2019-22-APN-PRES # SENASA - Veterinary products: prohibition of processing, distribution, import, use and possession.* 2019.

101. Pan American Health Organization (PAHO) and World Health Organization (WHO), *Epidemiological Alert - Enterobacteriaceae with plasmid-mediated transferrable colistin resistance, public health indications in the Americas.* 2016.

102. Mendelson, M., A. Brink, J. Gouws, et al., *The One Health stewardship of colistin as an antibiotic of last resort for human health in South Africa.* Lancet Infectious Diseases, 2018. **18**(9): p. E288-E294.

103. Labuschagne, Q., N. Schellack, A. Gous, et al., *COLISTIN: adult and paediatric guideline for South Africa, 2016.* Southern African Journal of Infectious Diseases, 2016. **31**(1): p. 3-7.

104. South African Medicines Control Council, *MEDICINES AND RELATED SUBSTANCES ACT.* 2017.

105. Maalej, S.M., M.R. Meziou, F. Mahjoubi, et al., *Epidemiological study of Enterobacteriaceae resistance to colistin in Sfax (Tunisia).* Medecine Et Maladies Infectieuses, 2012. **42**(6): p. 256-263.
